# Blockade of V‐domain immunoglobulin suppressor of T‐cell activation reprograms tumour‐associated macrophages and improves efficacy of PD‐1 inhibitor in gastric cancer

**DOI:** 10.1002/ctm2.1578

**Published:** 2024-02-15

**Authors:** Yifan Cao, Kuan Yu, Zihao Zhang, Yun Gu, Yichao Gu, Wandi Li, Weijuan Zhang, Zhenbin Shen, Jiejie Xu, Jing Qin

**Affiliations:** ^1^ Department of General Surgery Zhongshan Hospital, Fudan University Shanghai China; ^2^ Department of Immunology School of Basic Medical Sciences, Fudan University Shanghai China; ^3^ Department of Biochemistry and Molecular Biology School of Basic Medical Sciences, Fudan University Shanghai China

**Keywords:** gastric cancer, immunotherapy, PD‐1, tumour‐associated macrophages, VISTA

## Abstract

**Background and aims:**

In gastric cancer, the response rate of programmed cell death protein‐1 (PD‐1) inhibitor is far from satisfactory, indicating additional nonredundant pathways might hamper antitumour immunity. V‐domain immunoglobulin suppressor of T‐cell activation (VISTA) has been reported in several malignancies as a novel immune‐checkpoint. Nevertheless, the role of VISTA in gastric cancer still remains obscure. Our purpose is to explore the clinical significance and potential mechanism of VISTA in affecting gastric cancer patients’ survival and immunotherapeutic responsiveness.

**Methods:**

Our study recruited eight independent cohorts with a total of 1403 gastric cancer patients. Immunohistochemistry, multiplex immunofluorescence, flow cytometry or intracellular flow cytometry, quantitative polymerase chain reaction, western blotting, fluorescence‐activated cell sorting, magnetic‐activated cell sorting, smart‐seq2, in vitro cell co‐culture and ex vivo tumour inhibition assays were applied to investigate the clinical significance and potential mechanism of VISTA in gastric cancer.

**Results:**

VISTA was predominantly expressed on tumour‐associated macrophages (TAMs), and indicated poor clinical outcomes and inferior immunotherapeutic responsiveness. VISTA^+^ TAMs showed a mixed phenotype. Co‐culture of TAMs and CD8^+^ T cells indicated that VISTA^+^ TAMs attenuated effective function of CD8^+^ T cells. Blockade of VISTA reprogrammed TAMs to a proinflammatory phenotype, reactivated CD8^+^ T cells and promoted apoptosis of tumour cells. Moreover, blockade of VISTA could also enhance the efficacy of PD‐1 inhibitor, suggesting that blockade of VISTA might synergise with PD‐1 inhibitor in gastric cancer.

**Conclusions:**

Our data revealed that VISTA was an immune‐checkpoint associated with immunotherapeutic resistance. Blockade of VISTA reprogrammed TAMs, promoted T‐cell‐mediated antitumour immunity, and enhanced efficacy of PD‐1 inhibitor, which might have implications in the treatment of gastric cancer.

## Graphical abstract

VISTA indicated poor clinical outcomes and inferior immunotherapeutic responsivenessin gastric cancer.

VISTA was predominantly expressed on tumor‐associated macrophages (TAMs) in gastriccancer.

Blockade of VISTA reprogrammed TAMs, promoted T cell‐mediated antitumor immunity, andenhanced efficacy of PD‐1 inhibitor in gastric cancer.

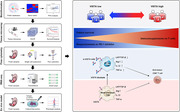

## 1 INTRODUCTION

Gastric cancer is a major global health burden.^1^ Worldwide, gastric cancer is the fifth most diagnosed cancer, with over 1,000,000 estimated new cases annually. Due to its covert early symptoms, gastric cancer is frequently diagnosed at advanced‐stage, making it the fourth most common cause of malignancy‐associated deaths.^2^ In recent years, although progress has been made in adjuvant treatment, the prognosis of advanced stage gastric cancer patients still remains dismal and requires to be improved.^3^

Stepping into the 21st century, cancer immunotherapy with immune‐checkpoint blockade (ICB) has emerged as a powerful weapon against cancer and led to important clinical advances.^4^ Antibodies targeting immune‐checkpoint receptors of B7 family, cytotoxic T‐lymphocyte‐associated protein‐4 (CTLA‐4) and programmed cell death protein‐1 (PD‐1), have shown durable response in a series of previously refractory malignancies, which are considered as a breakthrough in the treatment of cancer.^4^, ^5^ In 2021, nivolumab (*Opdivo*, Bristol‐Myers Squibb Company) was approved as first‐line treatment agents for advanced or metastatic gastric cancer and gastroesophageal junction cancer in the United States and the People's Republic of China. Despite this success, the response rate of ICB was generally less than 30%, and single‐agent PD‐1 inhibitors reported response rates of only 10%–17% for unselected patients with metastatic gastroesophageal cancer,^6^, ^7^ indicating additional non‐redundant immunomodulation pathways might attenuate antitumour immunity in gastric cancer.

As an immune‐checkpoint of B7 family.^8^, ^9^ V‐domain immunoglobulin suppressor of T‐cell activation (VISTA) is expressed on several types of immune cells, including macrophages, dendritic cells (DCs), naïve CD4^+^ T cells and Foxp3^+^CD4^+^ regulatory T (T_reg_) cells, with the highest expression found on myeloid cells.^8^, ^10^ Different from PD‐1 or CTLA‐4, which controls T‐cell‐mediated immunity and antagonizes T‐cell receptor signalling,^11^, ^12^ VISTA plays a more profound role in modulating myeloid cell‐mediated immune responses.^13^ Therefore, VISTA could serve as a potential immunotherapeutic target.^14^, ^15^ Phase I/II clinical trials of VISTA inhibitors (JNJ‐61610588; CI‐8993; CA‐170) are currently ongoing.^16^ Nevertheless, knowledge about the role of VISTA in gastric cancer is still limited. Whether VISTA reprograms macrophages and indirectly controls tumour‐specific T‐cell responses in gastric cancer has not been elucidated, either.

Our study revealed a potential role of VISTA in modulating the phenotype of tumour‐associated macrophages (TAMs) and orchestrating immunotherapeutic resistance. Blockade of VISTA abolished the immunosuppressive function of TAMs and led to a T‐cell‐effective tumour microenvironment that drove an efficient antitumour response. We propose VISTA as a new immunotherapeutic target in gastric cancer.

## 2 MATERIALS AND METHODS

### 2.1 Patients and specimens

Our study enrolled eight independent datasets with a total of 1403 gastric cancer patients. Discovery Dataset (from The Cancer Genome Atlas‐Stomach Adenocarcinoma [TCGA‐STAD], *n* = 407),^17^ External Validation Dataset (from GSE62254, the Asian Cancer Research Group [ACRG], *n* = 300),^18^ Expansion Dataset #1 (from Samsung Medical Center Sungkyunkwan University [SKKU‐SMC], *n* = 61),^19^ Expansion Dataset #3 (GSE134520, *n* = 9)^20^ and Expansion Dataset #4 (from Peking University‐short for single‐cell RNA‐seq (scRNA‐seq) data visualization and analyzation [PKU‐scDVA], *n* = 10)^21^ were public datasets. Internal Validation Dataset (T13‐564, *n* = 496),^22^ Expansion Dataset #2 (T19‐0097, *n* = 60)^23^ and Expansion Dataset #5 (Experimental Cohort [EXPC], *n* = 60) were our own datasets and were recruited from Zhongshan Hospital Fudan University (FDU‐ZSH). Basic information of data, data resources, patient inclusion and exclusion criteria were available (Figure S1 and Table S1).

### 2.2 Immunohistochemistry and multiplex immunofluorescence

The construction of tissue microarray (TMA) was documented (Supporting Information S1). The TMAs were heated at 60°C for 6 h, then immersed in xylene (three times, 15 min each) and alcohol (100%, 95%, 85%, 75%), and rinsed with Tris Buffered Saline with Tween 20 (TBST) (three times, 10 min each). Antigen retrieval was performed with Ethylene Diamine Tetraacetic Acid (EDTA) or Citrate antigen retrieval solution (ZSGB‐BIO). Endogenous peroxidase was blocked by endogenous peroxidase blocking buffer (ZSGB‐BIO) at 37°C for 30 min. Then, Normal Goat Serum (ZSGB‐BIO) was used to eliminate nonspecific reactions. Subsequently, the TMAs were incubated with primary antibody (Table S2) at 4°C overnight. Negative controls were treated identically with the primary antibody abandoned. After washing with TBST (three times, 10 min each), the TMAs were incubated with Horseradish Peroxidase (HRP)‐conjugated secondary antibody (ZSGB‐BIO) at 37°C for 20 min. For immunohistochemistry (IHC) staining, the TMAs were subsequently stained with 3,3'‐diaminobenzidine (DAB), counterstained with haematoxylin, then dehydrated and finally mounted with neutral resins and coverslip. For multiplex immunofluorescence (MxIF) staining, after incubation with HRP‐conjugated secondary antibody, the TMAs were immersed in Neon TSA fluorescent dye at room temperature for 10 min and washed with TBST (three times, 3 min each). The TMA slides underwent repeated steps mentioned above since antigen retrieval to staining with fluorescent dye. Then, the TMA slides were stained with 4',6‐diamidino‐2‐phenylindole (DAPI) at room temperature for 5 min. Eventually, the TMAs were sealed with anti‐fluorescence quenching sealant and coverslip.

### 2.3 Analysis of VISTA expression in gastric cancer

Expression of VISTA protein was scored in a blinded fashion. A detailed description of the IHC scoring system, together with representative images, scoring results and prognostic power analysis, was provided (Supporting Information S2 and Figures S2–S4).

### 2.4 Fluorescence‐activated cell sorting, magnetic‐activated cell sorting and RNA‐seq

Before fluorescence‐activated cell sorting (FACS), single‐cell suspensions derived from eight freshly resected gastric cancer tissues were treated with Percoll (Cytiva) to isolate mononuclear cells. VISTA^+^ TAMs (CD45^+^CD14^+^VISTA^+^) and VISTA^−^ TAMs (CD45^+^CD14^+^VISTA^−^) were subsequently isolated from these purified mononuclear cells by MoFlo XDP (Beckman Coulter) and immediately lysed in cell lysis buffer (10kgenomics) per 500 cells (Figure 4A). Before magnetic‐activated cell sorting (MACS), single‐cell suspensions derived from five freshly resected gastric cancer tissues were treated with IgG_2B_ (isotype, 15 μg/mL, R&D Systems) or anti‐human VISTA antibody (α‐VISTA, 15 μg/mL, R&D Systems) for 12 h at 37°C. Percoll (Cytiva) was subsequently applied to separate mononuclear cells. IgG_2B_‐treated or α‐VISTA‐treated TAMs were isolated from these purified mononuclear cells by human CD14 selection kit (MojoSort, BioLegend) and immediately lysed in cell lysis buffer (10kgenomics) per 1000 cells (Figure 5A). cDNA libraries were established by standard smart‐seq2 protocols.^24^ mRNA profiles were generated by NovaSeq 6000 Sequencing System (Illumina). RNA‐seq data were aligned to human transcriptome with use of HISAT2 software. Differentially expressed genes (DEGs) were analysed by R software (v4.1.2) and the DESeq2 package (v1.34.0). The Gene Ontology (GO) analysis was performed with DAVID bioinformatics resources (https://david.ncifcrf.gov/tools.jsp).^25^ Gene set enrichment analysis was performed for gene functional annotation. Detailed information about the antibodies, software and algorithms in this study was listed (Tables S2 and S3).

### 2.5 Cell lines

The human gastric cancer AGS cell line was obtained from QuiCell Biotechnology. AGS cells were mycoplasma free and cultured in Ham's F‐12K (Kaighn's) Medium (QuiCell), which was supplemented with 10% foetal bovine serum (FBS, Gibco), 100 U/mL penicillin and 100 μg/mL streptomycin (Pen/Strep, Gibco) under 5% CO_2_ at 37°C.

### 2.6 Quantitative polymerase chain reaction

TAMs and tumour‐infiltrating CD8^+^ T cells were isolated from fresh gastric cancer tissues by human CD14 and CD8 nanobeads (MojoSort, BioLegend), respectively. Total RNA from TAMs, tumour‐infiltrating CD8^+^ T cells and AGS cell line was extracted by FastPure Total RNA Isolation Kit (Vazyme Biotech). RNA quantity and quality were determined with use of DS‐11 Spectrophotometer (DeNovix). RNA was transcribed into cDNA by PrimeScript RT reagent Kit (TaKaRa). Quantitative polymerase chain reaction (qPCR) was performed on QuantStudio5 (Applied Biosystems) with use of SYBR Green dye (TaKaRa). The primers used in this study were also listed (Table S4). At least three independent experiments were repeated for one sample. Relative transcript levels were estimated by means of ΔΔCt method.

### 2.7 Western blot

RIPA lysis buffer (Epizyme) was used to lyse cells. After centrifugation, the supernatant was collected in a clean tube. BCA Protein Assay Kit (Epizyme) was applied to quantify protein concentrations. Subsequently, loading buffer (Epizyme) was mixed with supernatant and heated. Samples of equal amounts were separated by 10% PAGE Gel Fast Preparation Kit (Epizyme) and then transferred onto polyvinylidene difluoride (PVDF) membranes (Millipore). PVDF membranes were blocked with skimmed milk powder (Servicebio) dissolved in TBST at room temperature for 2 h. The membranes were incubated with primary antibodies against VISTA (1:1000, CST) and GAPDH (1:10 000, CST; Table S2) at 4°C overnight. GAPDH was used as control. After incubation with secondary antibodies (1:5000, Servicebio), the signals were visualised by the ECL system (Clinx Science Instruments). Experiments were performed in triplicate.

### 2.8 In vitro TAM/T‐cell co‐culture system

Ficoll (GE Healthcare) was applied to separate human PBMCs. CD8^+^ T cells were isolated from purified PBMCs or tumour tissue‐digested single‐cell suspensions by human CD8 nanobeads (MojoSort, BioLegend). During a 4‐day incubation, bead‐purified peripheral CD8^+^ T cells (1 × 10^5^ cells/well in 24‐well plates) were co‐cultured with IgG_2B_‐treated TAMs or α‐VISTA‐treated TAMs isolated from tumour tissues in 1 mL Roswell Park Memorial Institute (RPMI) 1640 (Gibco) with 10% FBS (Gibco), rhIL‐2 (80 ng/mL, BioLegend), anti‐CD3 (2 μg/mL, BioLegend) and anti‐CD28 (1 μg/mL, BioLegend) antibodies (Figure 6G‐H). After 4‐day incubation, CD8^+^ T cells were harvested for flow cytometry (FC) or intracellular flow cytometry (ICFC).

### 2.9 Ex vivo tumour inhibition assay

Single‐cell suspensions derived from 10 freshly resected gastric cancer tissues were randomly divided into four treatment groups (Figure 7A): isotype control (IgG_2B_, 15 μg/mL, R&D Systems; IgG_4_, 10 μg/mL, BioLegend); VISTA blockade subgroup (α‐VISTA antibody, 15 μg/mL, R&D Systems; IgG_4_, 10 μg/mL, BioLegend); PD‐1 blockade subgroup (camrelizumab, 10 μg/mL, Suncadia Biopharmaceuticals; IgG_2B_, 15 μg/mL, R&D Systems); dual blockade subgroup (α‐VISTA antibody, 15 μg/mL, R&D Systems; camrelizumab, 10 μg/mL, Suncadia Biopharmaceuticals). Each treatment group was cultured in RPMI 1640 with 10% FBS for 12 h at 37°C. In another CD8^+^ T‐cell‐deprived ex vivo tumour inhibition model (Figure 7B), CD8^+^ T cells were depleted by human CD8 nanobeads (MojoSort, BioLegend). Single‐cell suspensions were cultured in 1 mL RPMI 1640 with 10% FBS. Then, the cells were cultured with α‐VISTA antibody (15 μg/mL, R&D Systems) or IgG_2B_ (15 μg/mL, R&D Systems) for 12 h at 37°C. After overnight culture, the cells were harvested for phenotype analysis by FC/ICFC. Detailed information about the antibodies was supplemented (Table S2).

### 2.10 Flow cytometry and intracellular flow cytometry

Freshly resected gastric cancer tissues were collected, digested and incubated with GolgiStop (BD Biosciences) at 37°C for 2 h. Then, the single‐cell suspensions were lysed with lysing buffer (BD Biosciences), protected from light, at room temperature for 15 min. The cells were stained with FVS510 (BD Biosciences) to determine live/dead cells. Afterwards, human Fc Block (BD Biosciences) was applied to block Fc receptor. Cells were stained for surface markers at 4°C for 30 min and protected from light. If necessary, cells were fixed and permeabilized with Fixation and Permeabilization Solution (BD Biosciences) at 4°C for 20 min. Intracellular cytokine staining was performed at 4°C for 30 min and protected from light. Stained cells were ultimately washed and resuspended in stain buffer (BD Biosciences). Flow cytometry (FC) or intracellular flow cytometry (ICFC) was performed by FACSCelesta or FACSAria III (BD Biosciences). Data were analysed by FlowJo (Tree Star).

### 2.11 Statistical analyses

Statistical analyses were conducted with SPSS v21.0 (International Business Machines), GraphPad Prism v9.0.0 (GraphPad Software) and Stata v14.2 (StataCorp). Pearson's *χ* ^2^ test or Fisher's exact test was applied for categorical variables, whereas Student's *t*‐test, Mann–Whitney *U*‐test, analysis of variance (ANOVA) or Kruskal–Wallis test was employed for continuous variables. Log‐rank test or multivariate analysis based on Cox proportional hazards method was performed to investigate the clinical significance. Detailed information about the software and algorithms used in this study was listed (Table S3). *p* < .05 was defined as statistically significant.

## 3 RESULTS

### 3.1 VSIR shows distinct expression pattern and predicts inferior immunotherapeutic responsiveness in gastric cancer

**FIGURE 1:**
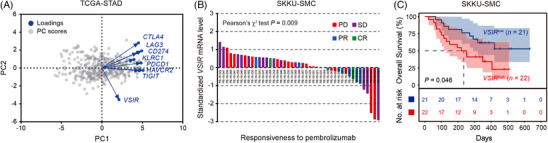
High expression of *VSIR* predicts inferior immunotherapeutic responsiveness in gastric cancer. (A) Principal component analysis (PCA) was applied to investigate the expression pattern of eight immune‐checkpoint‐associated genes (*VSIR* [VISTA], *HAVCR2* [TIM‐3], *TIGIT* [TIGIT], *KLRC1* [NKG2A], *CD274* [PD‐L1], *LAG3* [LAG‐3], *CTLA4* [CTLA‐4] and *PDCD1* [PD‐1]) within Discovery Dataset (The Cancer Genome Atlas‐Stomach Adenocarcinoma [TCGA‐STAD]). TCGA‐STAD was a public gastric cancer database including 407 gastric cancer patients, 375 of whom had comprehensive mRNA expression data (Figure S1), downloaded from National Cancer Institute GDC Data Portal (https://portal.gdc.cancer.gov/). (B) In Expansion Dataset #1 (Samsung Medical Center Sungkyunkwan University [SKKU‐SMC]), the patients stratified in *VSIR* ^high^ subgroup had significantly higher rate of progressive disease (PD)/stable disease (SD), compared with those in *VSIR* ^low^ subgroup after pembrolizumab‐based immunotherapy. In SKKU‐SMC cohort, gastric cancer patients were administered with a 30‐min intravenous infusion of pembrolizumab 200 mg every 3 weeks until documented disease progression, unacceptable toxicity, or up to 24 months. Tumour tissues were obtained before initiation of immunotherapy.^19^ SKKU‐SMC was a public gastric cancer immunotherapy database including 61 gastric cancer patients, 45 of whom had comprehensive mRNA expression data (Figure S1), downloaded from TIDE (http://tide.dfci.harvard.edu/). (C) In Expansion Dataset #1 (SKKU‐SMC), the patients stratified in *VSIR* ^high^ subgroup had poorer overall survival (OS) than those in *VSIR* ^low^ subgroup. SKKU‐SMC was a public gastric cancer immunotherapy database including 61 gastric cancer patients, 43 of whom had both mRNA data and survival data (Figure S1). Significance values were determined by Pearson's *χ* ^2^ test (B) or log‐rank test (C). The cut‐off value for classification of *VSIR* ^high^ subgroup and *VSIR* ^low^ subgroup was the median value. CR, complete response; CTLA‐4, cytotoxic T‐lymphocyte‐associated protein‐4; HAVCR2, hepatitis A virus cellular receptor 2; KLRC1, killer cell lectin like receptor C1; LAG‐3, lymphocyte activating gene‐3; NKG2A, natural killer group 2 member A; PD‐L1, programmed cell death 1‐ligand 1; PD‐1, programmed cell death protein‐1; PR, partial response; TIGIT, T‐cell immunoreceptor with Ig and ITIM domains; TIM‐3, T‐cell immunoglobulin domain and mucin domain‐3; VISTA, V‐domain immunoglobulin suppressor of T‐cell activation; VSIR, V‐set immunoregulatory receptor.

In Discovery Dataset (TCGA‐STAD), we investigated the expression pattern of eight immune‐checkpoint‐associated genes in gastric cancer: *VSIR* (VISTA),^26^ *HAVCR2* (TIM‐3),^27^ *TIGIT* (TIGIT),^28^ *KLRC1* (NKG2A),^29^ *CD274* (PD‐L1),^30^ *LAG3* (LAG‐3),^31^ *CTLA4* (CTLA‐4)^4^ and *PDCD1* (PD‐1),^32^ which were previously reported to be promising targets for immunotherapy. We found that the expression pattern of VISTA‐encoding gene *VSIR* was distinguished from those of other genes (Figure 1A), indicating *VSIR* (VISTA) might mediate an additional nonredundant immune‐checkpoint pathway in gastric cancer. Consequently, we included Expansion Dataset #1 from SKKU‐SMC and inspected the association between *VSIR* expression and patient responsiveness to pembrolizumab‐based immunotherapy (Figure S1). In SKKU‐SMC cohort, gastric cancer patients were administered with pembrolizumab. Tumour tissues were obtained before initiation of immunotherapy.^19^ Of note, we found after the application of pembrolizumab, the patients stratified in *VSIR* ^high^ subgroup had significantly higher rate of progressive disease (PD) or stable disease (SD), compared with those in *VSIR* ^low^ subgroup (Figure 1B). Furthermore, the patients stratified in *VSIR* ^high^ subgroup also underwent significantly poorer overall survival (OS) than those in *VSIR* ^low^ subgroup after pembrolizumab‐based immunotherapy (Figure 1C). Conclusively, these results indicated that high expression of *VSIR* could predict inferior immunotherapeutic responsiveness in gastric cancer.

### 3.2 Expression of VISTA correlates with adverse survival outcomes in gastric cancer

**FIGURE 2:**
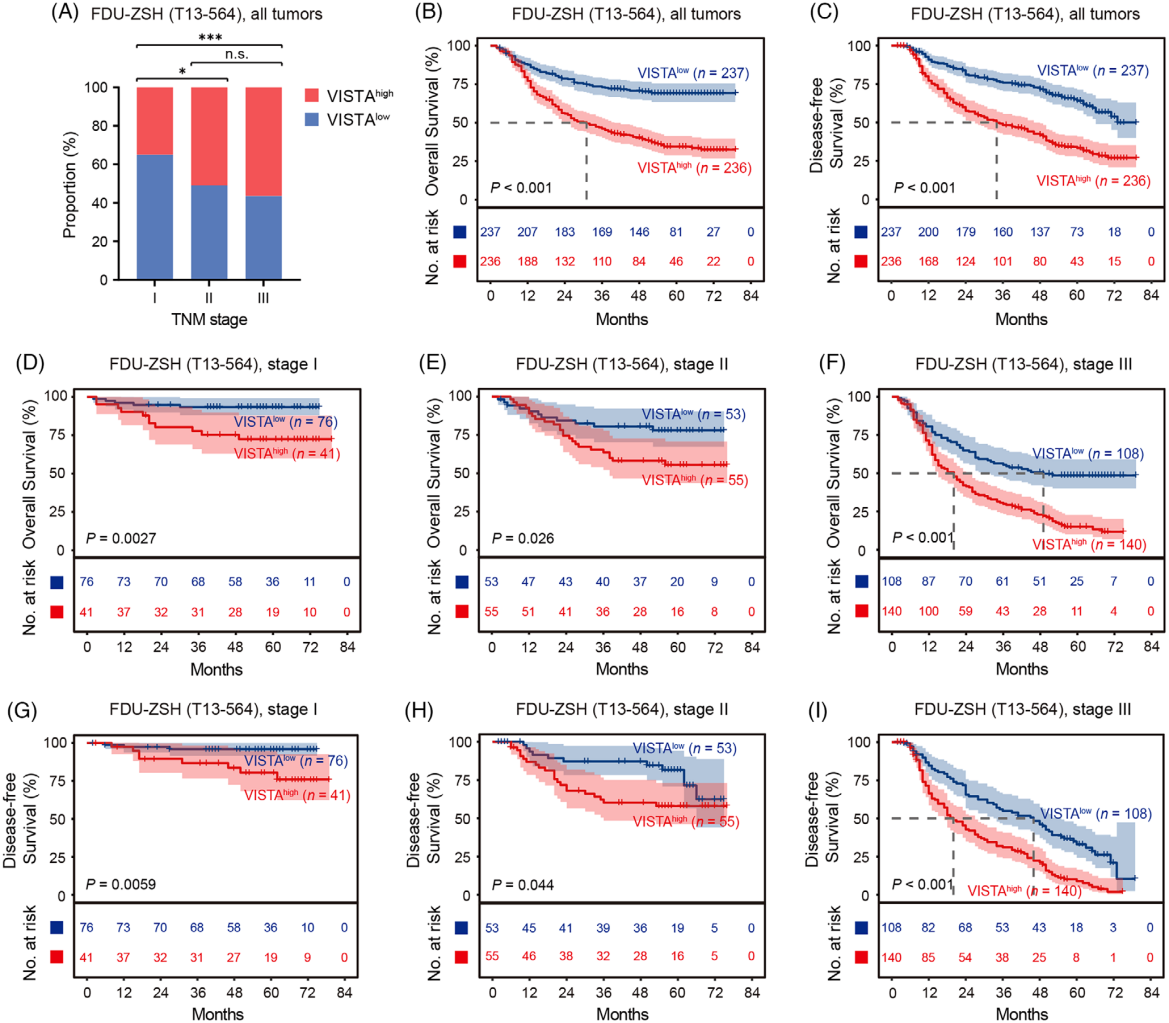
High expression of V‐domain immunoglobulin suppressor of T‐cell activation (VISTA) predicts poor survival outcomes in gastric cancer. (A) In Internal Validation Dataset (Zhongshan Hospital Fudan University [FDU‐ZSH] T13‐564), higher proportion of stage II and stage III patients were stratified into VISTA^high^ subgroup than stage I patients. FDU‐ZSH (T13‐564) was our own gastric cancer database including 496 gastric cancer patients, 473 of whom had resectable disease and comprehensive survival data (Figure S1). (B–I) In Internal Validation Dataset (FDU‐ZSH T13‐564), high expression of VISTA predicted poor overall survival (OS) and disease‐free survival (DFS) in any tumour‐node‐metastasis (TNM) stage. Significance values were determined by Pearson's *χ* ^2^ test followed by Bonferroni multiple comparison test (A) or log‐rank test (B–I). ^*^ *p* < .05, ^***^ *p* < .001, and n.s. refers to not significant. The cut‐off value for classification of VISTA^high^ subgroup and VISTA^low^ subgroup was the median value.

IHC showed that the expression of VISTA in tumour tissues was significantly higher than that in peritumour tissues (Figure S2). Moreover, VISTA was predominantly expressed in tumour stroma, whereas malignant epithelial showed almost no expression of VISTA (Figure S3). Consequently, we focused on the expression of VISTA in tumour tissues in the following study (Figure S4). Interestingly, we found that higher proportion of stage II and stage III patients were stratified into VISTA^high^ subgroup, compared with stage I patients (Figure 2A), indicating that VISTA was possibly associated with tumour progression. The patients stratified in VISTA^high^ subgroup had significantly poorer OS and disease‐free survival (DFS) than the patients in VISTA^low^ subgroup (Figure 2B,C). Notably, high expression of VISTA predicted poorer OS and DFS in any tumour‐node‐metastasis (TNM) stage (Figure 2D–I). These results showed that high VISTA expression indicated adverse survival outcomes in gastric cancer, which was independent of TNM stage. Furthermore, according to univariate analysis, size, Lauren classification, TNM stage and VISTA expression showed significant prognostic value in patient OS, while sex, size, Lauren classification, TNM stage and VISTA expression for DFS (Table 1). However, multivariate analysis showed that age, TNM stage and VISTA expression were significant variables for OS, and sex, Lauren classification, TNM stage and VISTA expression for DFS (Table 1). To compare the prognostic power between these significant variables, we applied time‐dependent area under the curve (AUC). Interestingly, TNM stage showed highest AUC in predicting either OS or DFS, and VISTA expression was inferior to TNM stage but superior to age, sex or Lauren classification (Figure S5). Conclusively, these results emphasised the clinical significance of VISTA and characterised VISTA as an independent adverse prognosticator in gastric cancer.

**TABLE 1:** Univariate and multivariate analyses for overall survival and disease‐free survival in Zhongshan Hospital Fudan University (FDU‐ZSH) (T13‐564).

	Overall survival				Disease‐free survival			
	Univariate		Multivariate		Univariate		Multivariate	
Factors	HR (95% CI)	*p*	HR (95% CI)	*p*	HR (95% CI)	*p*	HR (95% CI)	*p*
Age (years)		.10		**.037** ^a^		.21		.13
<60	1.00 (reference)		1.00 (reference)		1.00 (reference)		1.00 (reference)	
≥60	1.24 (.96–1.62)		1.33 (1.02–1.75)		1.18 (.91–1.54)		1.23 (.94–1.62)	
Sex		.079		.060		**.040**		**.023**
Female	1.00 (reference)		1.00 (reference)		1.00 (reference)		1.00 (reference)	
Male	.78 (.59–1.03)		.76 (.58–1.01)		.74 (.56–.99)		.72 (.54–.96)	
Size (cm)		**<.001**		.55		**<.001**		.10
<4	1.00 (reference)		1.00 (reference)		1.00 (reference)		1.00 (reference)	
≥4	1.68 (1.29–2.19)		1.09 (.83–1.43)		2.06 (1.58–2.70)		1.26 (.96–1.66)	
Lauren classification		**.005**		.070		**.001**		**.029**
Intestinal type	1.00 (reference)		1.00 (reference)		1.00 (reference)		1.00 (reference)	
Diffuse type	1.46 (1.12–1.90)		1.29 (.98–1.69)		1.59 (1.22–2.08)		1.36 (1.03–1.78)	
TNM stage^b^	2.80 (2.24–3.51)	**<.001**	2.65 (2.10–3.34)	**<.001**	3.84 (3.00–4.92)	**<.001**	3.68 (2.85–4.76)	**<.001**
VISTA expression		**<.001**		**<.001**		**<.001**		**<.001**
Low	1.00 (reference)		1.00 (reference)		1.00 (reference)		1.00 (reference)	
High	2.69 (2.02–3.56)		2.36 (1.78–3.14)		2.41 (1.83–3.17)		2.17 (1.64–2.86)	

Abbreviations: CI, confidence interval; HR, hazard ratio; TNM, tumour‐node‐metastasis; VISTA, V‐domain immunoglobulin suppressor of T‐cell activation.

^a^ *p* < .05 marked in bold font shows statistical significance.

^b^ Modelled as a continuous variable.

### 3.3 VISTA is predominantly expressed on TAMs in gastric cancer

**FIGURE 3:**
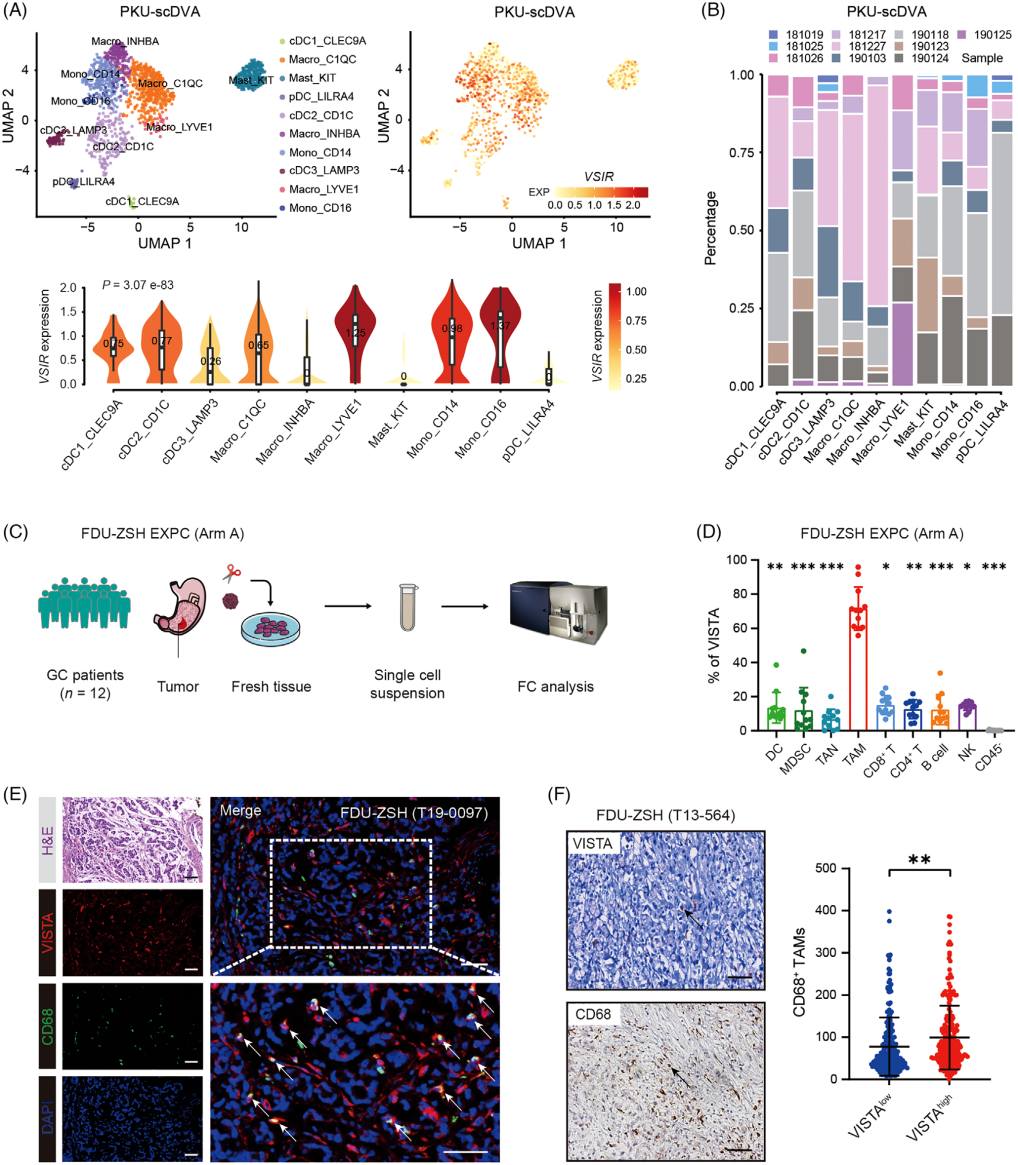
V‐domain immunoglobulin suppressor of T‐cell activation (VISTA) is predominantly expressed on tumour‐associated macrophages (TAMs) in gastric cancer. (A and B) According to Expansion Dataset #4 (Peking University‐short for single‐cell RNA‐seq data visualisation and analysation [PKU‐scDVA]), *VSIR* was potentially expressed on monocytes/macrophages in gastric cancer. PKU‐scDVA was a public single‐cell RNA‐seq database and contained 10 gastric cancer patients’ single‐cell RNA‐seq data (Figure S1), which were available at scDVA online website (http://panmyeloid.cancer‐pku.cn/). (C and D) Twelve fresh gastric cancer samples (Zhongshan Hospital Fudan University [FDU‐ZSH] Experimental Cohort [EXPC] Arm A) were collected and performed with flow cytometry (FC)/intracellular flow cytometry (ICFC). VISTA was primarily expressed on TAMs. Data in panel (D) are expressed as mean ± standard deviation. FDU‐ZSH EXPC (Arm A) was our own dataset including 12 gastric cancer patients. Fresh gastric cancer samples were collected from these 12 patients and performed with FC to investigate VISTA expression within different cell types (Figure S1). (E) In FDU‐ZSH (T19‐0097) cohort, multiplex immunofluorescence (MxIF) was performed to validate the co‐expression of VISTA and CD68 in gastric cancer. White arrowheads show VISTA^+^ TAMs. Haematoxylin and eosin (H&E) staining was also applied with the same tissue microarray (TMA). Scale bar represents 50 μm. FDU‐ZSH (T19‐0097) was our own dataset, including 60 gastric cancer patients (Figure S1). (F) In FDU‐ZSH (T13‐564) cohort, high expression of VISTA was correlated with increased infiltration of CD68^+^ TAMs. Data in panel (F) are expressed as mean ± standard deviation. Scale bar represents 50 μm. Significance values were determined by Kruskal–Wallis test followed by Dunn's multiple comparisons test (D) and Student's *t*‐test (F). ^**^ *p* < .01, ^***^ *p* < .001. UMAP, uniform manifold approximation and projection; *VSIR*, V‐set immunoregulatory receptor.

Since VISTA might inhibit antitumour immunity, we subsequently sought for the source of VISTA in gastric cancer. According to the scRNA‐seq data from Expansion Dataset #3 (GSE134520),^20^ we found *VSIR* was expressed on several kinds of cells, especially myeloid cells including macrophages, monocytes and DCs (Figure S6). Another scRNA‐seq database PKU‐scDVA^21^ also indicated that *VSIR* was predominantly expressed on monocytes/macrophages in gastric cancer (Figure 3A,B). Consequently, we collected 12 fresh gastric cancer samples from FDU‐ZSH (EXPC Arm A), and performed FC to validate the distribution of VISTA (Figure 3C). We found that VISTA was primarily expressed on TAMs (Figures 3D and S7). MxIF also verified the co‐expression of VISTA and CD68 in gastric cancer (Figure 3E). Since previous studies reported VISTA could also be expressed by CD8^+^ T cells and cancer cells,^33^ or expressed VISTA may be translocated onto the cell surface, where it contributes to suppression of T‐cell activity.^34^ VISTA can also be shed off the cell surface and secreted (40 kDa soluble VISTA).^35^, ^36^ Furthermore, we studied the *VSIR* mRNA level in TAMs to confirm that VISTA gene is expressed in these cells and that this protein did not come as a secreted protein from gastric cancer cells. Western blotting (WB) was also performed in TAMs to see what kind of VISTA expressed. According to qPCR, we found that TAMs showed significantly higher mRNA level of *VSIR* compared with CD8^+^ T cells or gastric cancer cells. According to WB, we also found that TAMs showed significantly higher expression of VISTA compared with CD8^+^ T cells or gastric cancer cells. Additionally, we found that TAMs expressed 55 kDa VISTA (Figure S8). Consequently, these results indicated that VISTA was expressed by TAMs and might serve as a receptor other than an extracellular domain proteolytically shed off the surface of other cells. Association between the expression of VISTA and CD68 was also validated in gastric cancer patient cohort (Figure 3F). Collectively, these findings suggested that VISTA was predominantly expressed on TAMs in gastric cancer.

### 3.4 VISTA^+^ TAMs show a mixed phenotype

**FIGURE 4:**
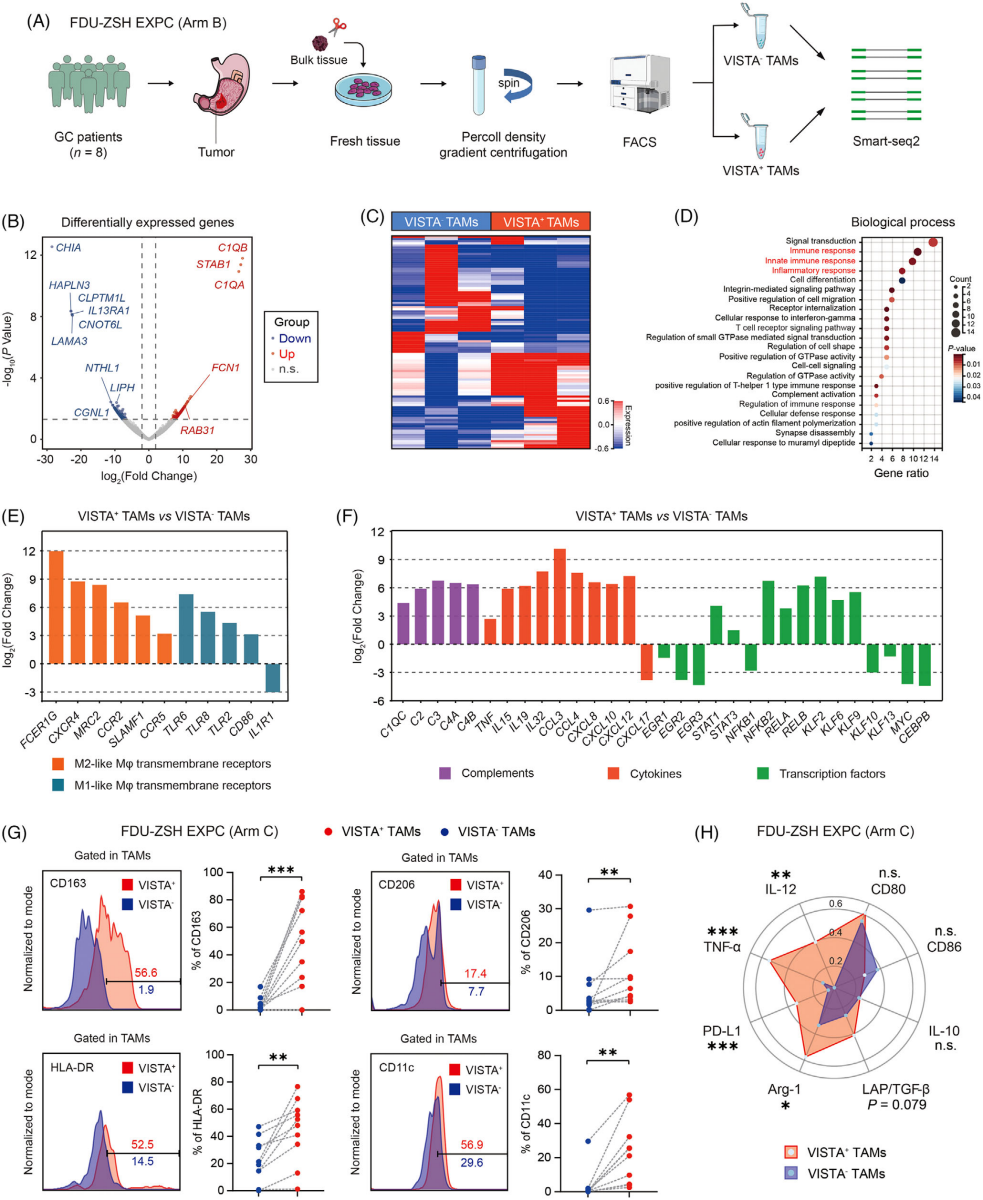
V‐domain immunoglobulin suppressor of T‐cell activation (VISTA^+^) tumour‐associated macrophages (TAMs) show a mixed phenotype in gastric cancer. (A) Eight fresh gastric cancer samples (Zhongshan Hospital Fudan University [FDU‐ZSH] Experimental Cohort [EXPC] Arm B) were collected and performed with fluorescence‐activated cell sorting (FACS) to sort eight pairs of VISTA^+^ TAMs and VISTA^−^ TAMs. After quality control (QC), three pairs of VISTA^+^ TAMs and VISTA^−^ TAMs were subsequently performed with smart‐seq2. FDU‐ZSH EXPC (Arm B) was our own dataset and contained 8 gastric cancer (GC) patients (Figure S1). (B and C) Differentially expressed genes (DEGs) of VISTA^+^ TAMs versus VISTA^−^ TAMs were detected in FDU‐ZSH EXPC (Arm B). (D) The Gene Ontology (GO) analysis based on the DEGs of VISTA^+^ TAMs versus VISTA^−^ TAMs indicated that the up‐regulated genes in VISTA^+^ TAMs might be associated with immune response, innate immune response and inflammatory response, with use of FDU‐ZSH EXPC (Arm B). (E and F) The gene profile of transmembrane receptors, cytokines and transcription factors, based on the DEGs of VISTA^+^ TAMs versus VISTA^−^ TAMs, characterised VISTA^+^ TAMs as a specific subset of TAM populations that exhibited a mixed phenotype of M1 and M2‐like TAMs. (G) In FDU‐ZSH EXPC (Arm C), FC analysis with 10 fresh GC samples validated that VISTA^+^ TAMs exhibited significantly higher expression of M2‐like marker CD163 and CD206, as well as M1‐like marker human leukocyte antigen (HLA)‐DR and CD11c. FDU‐ZSH EXPC (Arm C) was our own dataset and contained 10 GC patients (Figure S1). (H) In FDU‐ZSH EXPC (Arm C), VISTA^+^ TAMs showed higher expression of tumour necrosis factor‐α (TNF‐α), interleukin (IL)‐12, programmed cell death 1‐ligand 1 (PD‐L1), arginase (Arg)‐1 and latency‐associated peptide (LAP)/transforming growth factor (TGF)‐β (marginally insignificant), compared with VISTA^−^ TAMs. Significance values were determined by paired *t* test (G [CD163, HLA‐DR and CD11c] and H) and Wilcoxon matched‐pairs signed rank test (G [CD206]). ^*^ *p* < .05, ^**^ *p* < .01, ^***^ *p* < .001 and n.s. refers to not significant.

To inspect the characteristics of VISTA^+^ TAMs, we collected eight fresh gastric cancer samples from FDU‐ZSH (EXPC Arm B), and performed FACS to sort eight pairs of VISTA^+^ TAMs and VISTA^−^ TAMs. After quality control (QC), three pairs of VISTA^+^ TAMs and VISTA^−^ TAMs were subsequently performed with smart‐seq2 (Figure 4A). VISTA^+^ TAMs expressed significantly higher expression of genes such as *C1QB*, *STAB1*, *C1QA*, *FCN1* and *RAB31* (Figure 4B), and showed different gene profile compared with VISTA^−^ TAMs (Figure 4C). Notably, *C1QA* ^+^ macrophage simultaneously resembled the signatures for TAMs and showed both M1 and M2‐like phenotype,^37^ while *FCN1* ^+^ monocyte‐like precursors might develop into *C1QC* ^+^ TAMs, which exhibited enriched complement activation, antigen processing and presentation pathways. And these cells could interact with other immune cells, especially T‐cell subsets.^21^, ^38^ However, *STAB1* (Stabilin‐1) was an M2‐like macrophage marker indicating immunosuppressive phenotype.^39^, ^40^ The GO analysis indicated that the up‐regulated genes in VISTA^+^ TAMs might be associated with immune response, innate immune response and inflammatory response (Figure 4D). Moreover, we found that the gene expression profile of transmembrane receptors, cytokines and transcription factors characterised VISTA^+^ TAMs as a specific subset of TAM populations that exhibited a mixed phenotype of M1 and M2‐like TAMs (Figure 4E,F), which was consistent with a previous study indicating M1 and M2 signatures did not necessarily exclude each other and often coexisted in TAMs.^41^ FC/ICFC analysis with fresh gastric cancer samples (FDU‐ZSH EXPC Arm C) also validated that VISTA^+^ TAMs exhibited significantly higher expression of M2‐like marker CD163 and CD206, as well as M1‐like marker human leukocyte antigen (HLA)‐DR and CD11c (Figure 4G). Moreover, we found that VISTA^+^ TAMs also showed higher expression of tumour necrosis factor‐α (TNF‐α), interleukin (IL)‐12, programmed cell death 1‐ligand 1 (PD‐L1), arginase (Arg)‐1 and latency‐associated peptide (LAP)/transforming growth factor (TGF)‐β, compared with VISTA^−^ TAMs (Figure 4H). Conclusively, these findings indicated that the phenotype of VISTA^+^ TAMs might be more complicated than the classical dichotomous phenotypes of M1 or M2‐like TAMs.

### 3.5 Blockade of VISTA reprograms TAMs in gastric cancer

**FIGURE 5:**
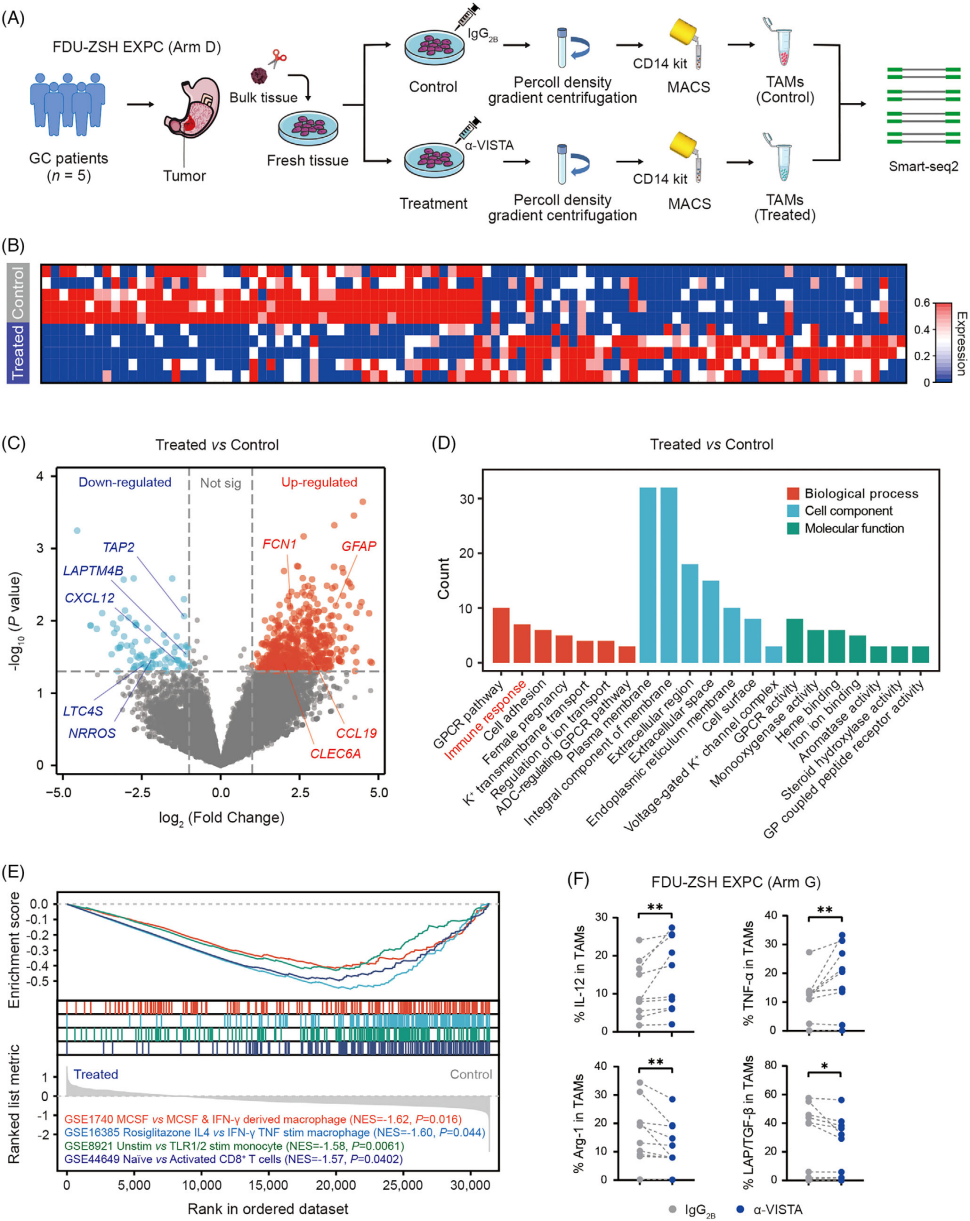
Blockade of V‐domain immunoglobulin suppressor of T‐cell activation (VISTA) reprograms tumour‐associated macrophages (TAMs) to a proinflammatory phenotype. (A) Five fresh gastric cancer (GC) samples (Zhongshan Hospital Fudan University [FDU‐ZSH] Experimental Cohort [EXPC] Arm D) were collected and digested into single‐cell suspensions. The single‐cell suspensions were incubated with IgG_2B_ (15 μg/mL, R&D Systems) or α‐VISTA antibody (15 μg/mL, R&D Systems) in RPMI 1640 medium (Gibco) containing 10% foetal bovine serum (FBS; Gibco) for 12 h at 37°C. Percoll (Cytiva) was subsequently applied to separate mononuclear cells. IgG_2B_‐treated TAMs and α‐VISTA antibody‐treated TAMs were isolated by CD14 magnetic‐activated cell sorting (MACS) kit, and performed with smart‐seq2. FDU‐ZSH EXPC (Arm D) was our own dataset and contained 5 GC patients (Figure S1). (B and C) Differentially expressed genes (DEGs) of α‐VISTA antibody‐treated TAMs versus IgG_2B_‐treated TAMs were detected within FDU‐ZSH EXPC (Arm D). (D and E) In FDU‐ZSH EXPC (Arm D), Gene Ontology (GO) and Kyoto Encyclopedia of Genes and Genomes (KEGG) analysis indicated that blockade of VISTA activated the genes associated with immune responses, especially the genes modulating proinflammatory phenotype of macrophages and CD8^+^ T‐cell activation. (F) According to flow cytometry (FC)/intracellular flow cytometry (ICFC) (FDU‐ZSH EXPC Arm G), blockade of VISTA significantly up‐regulated the expression of interleukin (IL)‐12 and tumour necrosis factor‐α (TNF‐α), yet down‐regulated the expression of arginase (Arg)‐1 and latency‐associated peptide (LAP)/transforming growth factor (TGF)‐β within TAMs. FDU‐ZSH EXPC (Arm G) was our own dataset, including 10 GC patients (Figure S1). Significance values were determined by Wilcoxon matched‐pairs signed rank test (F). ^*^ *p* < .05 and ^**^ *p* < .01.

Subsequently, we isolated TAMs from gastric cancer tissues after VISTA blockade or isotype treatment, and applied smart‐seq2 to inspect the impact of VISTA blockade on TAMs (Figure 5A). Interestingly, 651 DEGs were detected (Table S5). According to these DEGs, we constructed a VISTA^+^ TAM signature. Notably, higher VISTA^+^ TAM signature indicated significantly poorer OS in TCGA‐STAD database as well as ACRG (GSE62254) database (Figure S9). Furthermore, we found α‐VISTA‐treated TAMs showed decreased expression of *TAP2*, *LAPTM4B*, *CXCL12*, *LTC4S* and *NRROS*, yet increased expression of *FCN1*, *GFAP*, *CCL19* and *CLEC6A*, indicating a proinflammatory phenotype after blockade of VISTA (Figure 5B,C).^42^, ^43^, ^44^, ^45^, ^46^, ^47^, ^48^, ^49^, ^50^ Blockade of VISTA activated the genes associated with immune responses (Figure 5D), especially the genes modulating proinflammatory phenotype of macrophages and CD8^+^ T‐cell activation (Figure 5E). By means of FC/ICFC, we also validated that blockade of VISTA significantly up‐regulated the expression of TNF‐α and IL‐12, yet down‐regulated the expression of Arg‐1 and LAP/TGF‐β within TAMs (Figures 5F and S10). Of note, blockade of VISTA could not interfere with PD‐L1 expression on TAMs (Figure S10), indicating VISTA blockade might be non‐overlapping with PD‐1/PD‐L1 pathways, which was consistent with a previous study.^51^ Conclusively, these findings suggested that blockade of VISTA might reprogram TAMs to a proinflammatory phenotype.

### 3.6 VISTA^+^ TAMs attenuate effective function of CD8^+^ T cells

**FIGURE 6:**
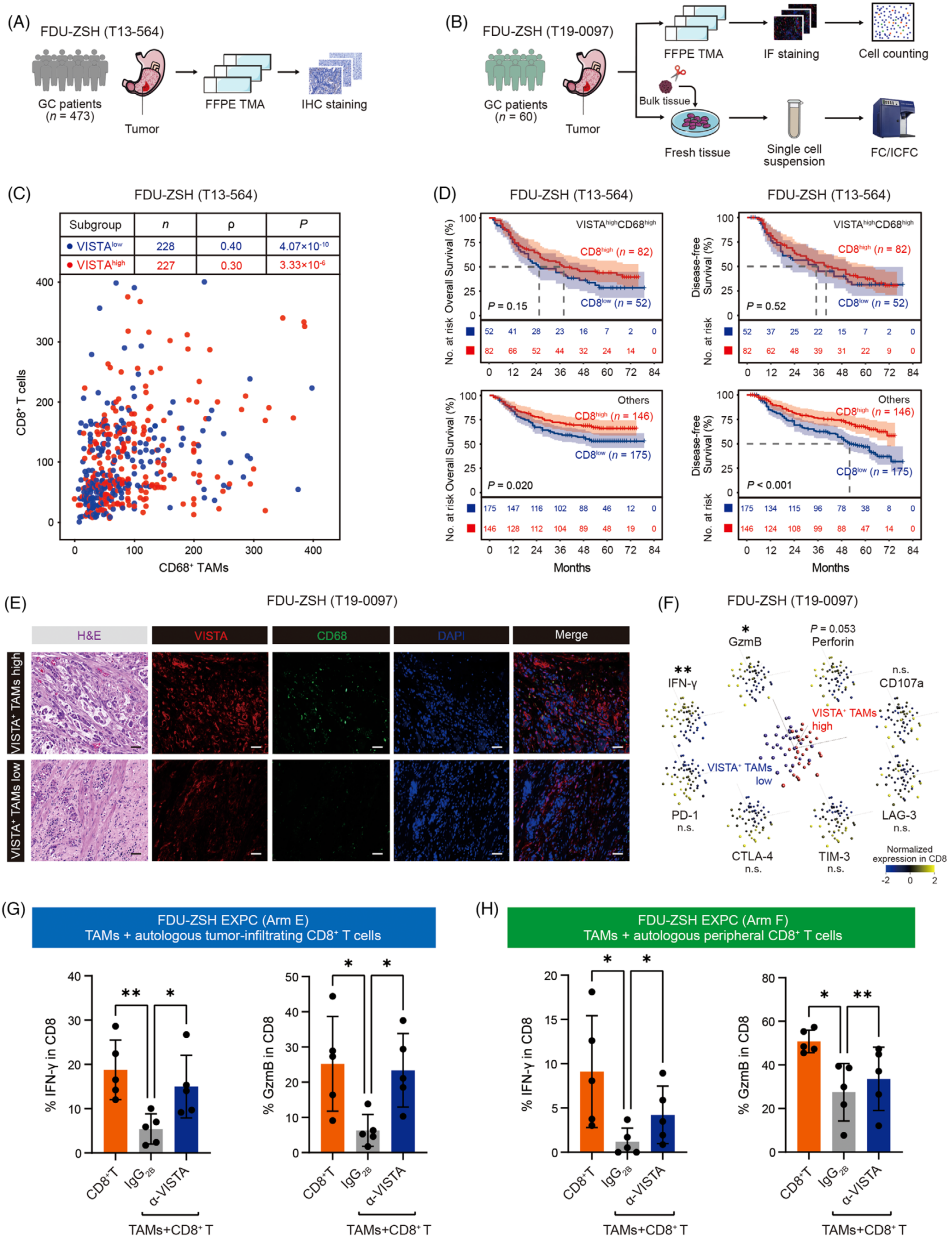
V‐domain immunoglobulin suppressor of T‐cell activation (VISTA^+^) tumour‐associated macrophages (TAMs) attenuate effective function of CD8^+^ T cells. (A) In Zhongshan Hospital Fudan University (FDU‐ZSH) (T13‐564) cohort, we performed immunohistochemistry (IHC) staining for VISTA, CD68 and CD8. FDU‐ZSH (T13‐564) was our own gastric cancer (GC) database including 496 GC patients, 473 of whom had resectable disease and comprehensive survival data (Figure S1). Eighteen patients had dot loss during the IHC staining, and finally 455 patients were assessable for VISTA, CD68 and CD8 in FDU‐ZSH (T13‐564) cohort. The cut‐off values for VISTA (6/HPF), CD68 (65/HPF) and CD8 (73/HPF) were median values according to IHC scores, respectively. (B) In FDU‐ZSH (T19‐0097) cohort, we performed flow cytometry (FC)/intracellular flow cytometry (ICFC) to analyse the phenotype of CD8^+^ T cells in tumour tissues, and we also conducted immunofluorescence (IF) staining for VISTA and CD68 with the tissue microarray (TMA) derived from the corresponding formalin‐fixed and paraffin‐embedded (FFPE) samples. FDU‐ZSH (T19‐0097) was our own dataset including 60 GC patients (Figure S1). (C) In FDU‐ZSH (T13‐564) cohort, infiltration of CD8^+^ T cells and CD68^+^ TAMs were analysed in VISTA^high^ and VISTA^low^ patients. The infiltration of CD8^+^ T cells were positively correlated with CD68^+^ TAMs, regardless of VISTA expression. (D) In VISTA^high^CD68^high^ patients, higher infiltration of CD8^+^ T cells could not predict better overall survival (OS) or disease‐free survival (DFS). However, higher infiltration of CD8^+^ T cells could significantly predict better OS and DFS in other patients. The cut‐off values for CD68 and CD8 were the median value, respectively. (E) Multiplex immunofluorescence (MxIF) staining for VISTA and CD68 with the FDU‐ZSH (T19‐0097) TMA. Red colour represents VISTA, green colour represents CD68 and blue colour represents DAPI. Haematoxylin and eosin (H&E) staining for the T19‐0097 TMA was also shown. Scale bar represents 50 μm. (F) According to the results from FC/ICFC and IF staining, which were performed within FDU‐ZSH (T19‐0097), principal component analysis (PCA) indicated that higher infiltration of VISTA^+^ TAMs was associated with decreased expression of interferon‐γ (IFN‐γ) and granzyme B (GzmB) within CD8^+^ T cells, while expression of Perforin showed marginal insignificance. Each symbol represents an individual patient. (G) Co‐culture with IgG_2B_‐treated TAMs could lead to decreased expression of IFN‐γ and GzmB within tumour‐infiltrating CD8^+^ T cells, while co‐culture with α‐VISTA‐treated TAMs showed restored cytotoxicity of CD8^+^ T cells. Data are expressed as mean ± standard deviation. FDU‐ZSH Experimental Cohort (EXPC) (Arm E) was our own dataset and contained 5 GC patients (Figure S1). (H) Co‐culture with IgG_2B_‐treated TAMs could lead to decreased expression of IFN‐γ and GzmB within peripheral CD8^+^ T cells, while co‐culture with α‐VISTA‐treated TAMs showed restored cytotoxicity of CD8^+^ T cells. Data are expressed as mean ± standard deviation. FDU‐ZSH EXPC (Arm F) was our own dataset and contained 5 GC patients (Figure S1). Significance values were determined by Spearman's correlation test (C), log‐rank test (D), Student's *t*‐test (F [Perforin, PD‐1 and TIM‐3]), Mann–Whitney *U*‐test (F [IFN‐γ, GzmB, CD107a, CTLA‐4 and LAG‐3]) and RM one‐way analysis of variance (ANOVA) followed by Dunnett's multiple comparisons test (G and H). CTLA‐4, cytotoxic T‐lymphocyte‐associated protein‐4; LAG‐3, lymphocyte activation gene‐3; MACS, magnetic‐activated cell sorting; PD‐1, programmed cell death protein‐1; TIM‐3, T‐cell immunoglobulin domain and mucin domain‐3.

Since VISTA^+^ TAMs might interfere with CD8^+^ T‐cell function (Figure 5E), we subsequently sought to inspect the potential impact of VISTA^+^ TAMs on CD8^+^ T cells. Based on FDU‐ZSH (T13‐564) cohort (Figure 6A), we found that infiltration of CD68^+^ TAMs was positively correlated with CD8^+^ T cells, regardless of VISTA expression (Figure 6C). Notably, increased infiltration of CD8^+^ T cells could predict better OS and DFS, except for the VISTA^high^CD68^high^ patients (Figure 6D), indicating VISTA^+^ TAMs might attenuate CD8^+^ T‐cell function. According to the results from FC/ICFC and MxIF (Figure 6B), we found that higher infiltration of VISTA^+^ TAMs was associated with decreased expression of interferon‐γ (IFN‐γ) and granzyme B (GzmB) within CD8^+^ T cells (Figure 6E,F). In TAM/T cell co‐culture system, blockade of VISTA mitigated the immunosuppressive effect of TAMs on CD8^+^ T cells (Figure 6G,H). Collectively, these results indicated that VISTA^+^ TAMs could possibly attenuate effective function of CD8^+^ T cells.

### 3.7 Blockade of VISTA reactivates effective function of CD8^+^ T cells and enhances efficacy of PD‐1 inhibitor

**FIGURE 7:**
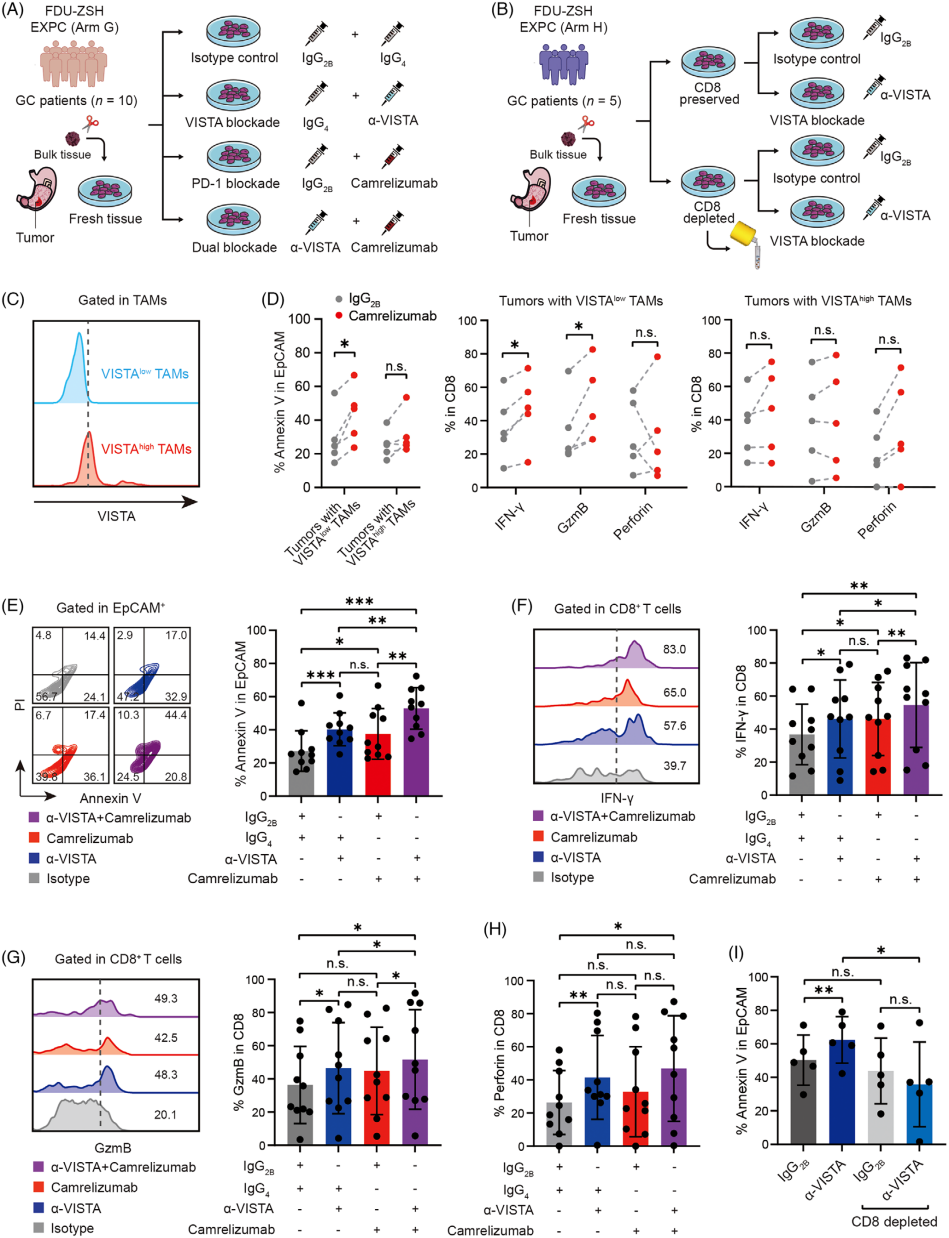
Blockade of V‐domain immunoglobulin suppressor of T‐cell activation (VISTA) enhances effective function of CD8^+^ T cells and promotes efficacy of programmed cell death protein‐1 (PD‐1) inhibitor. (A) An ex vivo tumour inhibition assay was established to investigate the potential impact of VISTA and/or PD‐1 blockade in gastric cancer (GC). Single‐cell suspensions derived from freshly resected GC tissues (Zhongshan Hospital Fudan University [FDU‐ZSH] Experimental Cohort [EXPC] Arm G) were randomly divided into four treatment groups: isotype control (IgG_2B_, 15 μg/mL, R&D Systems; IgG_4_, 10 μg/mL, BioLegend); VISTA blockade subgroup (α‐VISTA antibody, 15 μg/mL, R&D Systems; IgG_4_, 10 μg/mL, BioLegend); PD‐1 blockade subgroup (camrelizumab, 10 μg/mL, Suncadia Biopharmaceuticals; IgG_2B_, 15 μg/mL, R&D Systems); dual blockade subgroup (α‐VISTA antibody, 15 μg/mL, R&D Systems; camrelizumab, 10 μg/mL, Suncadia Biopharmaceuticals). Each treatment group was cultured in RPMI 1640 medium (Gibco) containing 10% foetal bovine serum (FBS; Gibco) for 12 h at 37°C. FDU‐ZSH EXPC (Arm G) was our own dataset and contained 10 GC patients (Figure S1). (B) In another CD8^+^ T‐cell‐deprived ex vivo tumour inhibition assay, CD8^+^ T cells were depleted from single‐cell suspensions (FDU‐ZSH EXPC Arm H) by human CD8 nanobeads (MojoSort, BioLegend). Single‐cell suspensions were cultured in 1 mL RPMI 1640 medium (Gibco) containing 10% FBS (Gibco). Then, the cells were cultured with α‐VISTA antibody (15 μg/mL, R&D Systems) or IgG_2B_ (15 μg/mL, R&D Systems) for 12 h at 37°C. After overnight culture, the cells were harvested for phenotype analysis by flow cytometry (FC)/intracellular flow cytometry (ICFC). FDU‐ZSH EXPC (Arm H) was our own dataset, including 5 GC patients (Figure S1). (C and D) In FDU‐ZSH EXPC (Arm G), blockade of PD‐1 showed increased apoptosis of tumour cells in the tumours infiltrated with VISTA^low^ TAMs, rather than VISTA^high^ TAMs. Similarly, elevated expression of interferon‐γ (IFN‐γ) and granzyme B (GzmB) within CD8^+^ T cells was observed in the tumours with VISTA^low^ TAMs rather than VISTA^high^ TAMs, after application of camrelizumab. (E) In FDU‐ZSH EXPC (Arm G), blockade of VISTA showed increased apoptosis of tumour cells. Combination of VISTA blockade and PD‐1 inhibitor camrelizumab showed more significant apoptosis of tumour cells, compared with VISTA blockade or camrelizumab alone. Data are expressed as mean ± standard deviation. (F–H) In FDU‐ZSH EXPC (Arm G), blockade of VISTA showed increased expression of IFN‐γ, GzmB and Perforin within CD8^+^ T cells. Combination of VISTA blockade and PD‐1 inhibitor camrelizumab showed more significant reactivation of CD8^+^ T‐cell‐effective function, compared with VISTA blockade or camrelizumab alone. Data are expressed as mean ± standard deviation. (I) In FDU‐ZSH EXPC (Arm H), the antitumour effect of VISTA blockade was abolished when CD8^+^ T cells were depleted from single‐cell suspensions, while the single‐cell suspensions with CD8^+^ T cells depletion alone did not show significant decrease in tumour cell apoptosis compared with those without CD8^+^ T cells depletion. Data are expressed as mean ± standard deviation. Significance values were determined by paired *t* test (D), RM one‐way analysis of variance (ANOVA) followed by Tukey's multiple comparisons test (E–H), or Sidak's multiple comparisons test (I). ^*^ *p* < .05, ^**^ *p* < .01, ^***^ *p* < .001 and n.s. refers to not significant. EpCAM, epithelial cell adhesion molecule.

Finally, we established an ex vivo tumour inhibition assay to investigate the potential impact of VISTA and/or PD‐1 blockade in gastric cancer (Figure 7A). Interestingly, we found blockade of PD‐1 showed increased apoptosis of tumour cells in the tumours infiltrated with VISTA^low^ TAMs, rather than VISTA^high^ TAMs. Similarly, after the application of camrelizumab, elevated expression of IFN‐γ and GzmB within CD8^+^ T cells was observed in the tumours with VISTA^low^ TAMs rather than VISTA^high^ TAMs (Figure 7C,D). These results indicated that VISTA^+^ TAMs were associated with reduced efficacy of PD‐1 inhibitor camrelizumab on promotion of tumour cell apoptosis and reactivation of CD8^+^ T‐cell‐effective function. Notably, blockade of VISTA showed increased apoptosis of tumour cells (Figure 7E), as well as increased expression of IFN‐γ, GzmB and Perforin within CD8^+^ T cells (Figure 7F–H). Moreover, combination of VISTA blockade and PD‐1 inhibitor camrelizumab showed more significant apoptosis of tumour cells and reactivation of CD8^+^ T cells, compared with VISTA blockade or camrelizumab alone, suggesting a potential synergistic effect of VISTA blockade and PD‐1 inhibitor camrelizumab in gastric cancer. To figure out the relation between promotion of tumour cell apoptosis and reactivation of CD8^+^ T cells, we established another ex vivo tumour inhibition assay with CD8^+^ T cells depleted (Figure 7B). The antitumour effect of VISTA blockade was abolished when CD8^+^ T cells were depleted, while the single‐cell suspensions with CD8^+^ T cells depletion alone did not show significant decrease in tumour cell apoptosis compared with those without CD8^+^ T cells depletion (Figure 7I), indicating blockade of VISTA might eventually reactivate effective function of CD8^+^ T cells and thus promote tumour cell apoptosis. Conclusively, these data suggested that blockade of VISTA could possibly reactivate effective function of CD8^+^ T cells and promote tumour cell apoptosis, and enhance efficacy of PD‐1 inhibitor in gastric cancer.

## 4 DISCUSSION

In recent years, several studies have reported VISTA as a potential prognosticator. Nevertheless, the conclusions remain controversial. In triple‐negative breast cancer (TNBC), VISTA indicated favourable prognosis and high infiltration of immune cells.^52^, ^53^ However, such published studies on TNBC were primarily bioinformatic ones and lacked further experiments to elucidate the distribution of VISTA or the mechanism of VISTA‐associated immuno‐oncology. In early‐stage esophageal adenocarcinoma, Loeser et al. found that VISTA positive tumours showed an obvious survival advantage compared with VISTA‐negative ones.^54^ However, the authors were concentrated on the expression of VISTA within tumour‐infiltrating T‐lymphocytes, and did not discuss VISTA on other cell types. Contrarily, Kuklinski et al. suggested that expression of VISTA was a negative prognosticator in primary cutaneous melanoma.^55^ To date, few studies have reported VISTA in gastric cancer. The clinical significance and underlying mechanism of VISTA‐associated immuno‐oncology in gastric cancer still remain elusive.

VISTA is reported to play a profound role in the modulation of immune responses.^8^, ^56^ Myeloid cells show the predominant expression of VISTA, but lymphoid cells such as T lymphocytes also express VISTA.^8^, ^56^ VISTA could serve as both receptor and ligand.^8^, ^56^ Receptors of VISTA require further investigation. One of the VISTA ligands is V‐set and immunoglobulin domain containing‐3 (VSIG‐3).^57^ Another ligand for VISTA is galectin‐9.^36^ Galectin‐9 can interact with VISTA on the surface of T cells, which results in T‐cell‐programmed death.^36^ In addition, recent study has reported that anti‐VISTA could boost antigen presentation pathways yet reduce myeloid cell‐mediated immunosuppression, indicating VISTA might play a crucial part in regulating immunosuppression function of myeloid cells.^58^

In current study, we observed that *VSIR* showed a unique expression pattern distinct from other immune‐checkpoint genes in gastric cancer, which indicated that not all gastric cancer might have the same immunotherapeutic responsiveness. Furthermore, we found that VISTA was preferentially expressed on TAMs in gastric cancer. Since previous studies reported VISTA could also be expressed by CD8^+^ T cells and cancer cells,^33^ or expressed VISTA may be translocated onto the cell surface, where it contributes to suppression of T‐cell activity.^34^ VISTA can also be shed off the cell surface and secreted (40 kDa soluble VISTA).^35^, ^36^ Furthermore, we studied the *VSIR* mRNA level in TAMs to confirm that VISTA gene was expressed in these cells and that this protein did not come as a secreted protein from gastric cancer cells. WB was also performed in TAMs to see what kind of VISTA expressed. According to qPCR, we found that TAMs showed significantly higher mRNA level of *VSIR* compared with CD8^+^ T cells or gastric cancer cells. According to WB, we also found that TAMs showed significantly higher expression of VISTA compared with CD8^+^ T cells or gastric cancer cells. Additionally, we found that TAMs expressed 55 kDa VISTA. Consequently, these results indicated that VISTA was expressed by TAMs and might serve as a receptor other than an extracellular domain proteolytically shed off the surface of other cells. According to FACS, smart‐seq2 and FC/ICFC, VISTA^+^ TAMs showed a mixed phenotype, more complicated than the classical dichotomous phenotypes of M1 or M2‐like TAMs, which might explain the paradoxical effect of VISTA in various malignancies to some extent. In view of recent studies on macrophage functions, growing evidence has proven that M1 and M2 phenotypes do not necessarily exclude each other but usually coexist. The mixed phenotype of macrophages relies on the balance of activatory and inhibitory activities, as well as the corresponding microenvironment.^41^ Our data suggested that these VISTA^+^ TAMs could attenuate effective function of CD8^+^ T cells and orchestrate immunotherapeutic resistance to PD‐1 inhibitor. Blockade of VISTA could reprogram TAMs to a proinflammatory phenotype, reactivate effective function of CD8^+^ T cells, promote tumour cell apoptosis, and enhance efficacy of PD‐1 inhibitor. These findings highlighted the significance of dual blockade of immune‐checkpoints, such as PD‐1/PD‐L1 and/or VISTA, might provide substantial clinical benefit for patients with gastric cancer.

Although our current study was a retrospective and exploratory one, which was primarily based on clinical specimens, we advocated for future studies to elucidate the mechanism of VISTA‐associated immunotherapeutic resistance, and validate whether VISTA could be targeted to reactivate antitumour responses in gastric cancer, within the framework of international, multi‐centred randomised clinical trials.

Conclusively, VISTA was predominantly expressed on TAMs, and indicated poor clinical outcomes and inferior immunotherapeutic responsiveness in gastric cancer. VISTA^+^ TAMs attenuated effective function of CD8^+^ T cells. Blockade of VISTA reprogrammed TAMs to a proinflammatory phenotype, reactivated effective function of CD8^+^ T cells, promoted tumour cell apoptosis, and enhanced efficacy of PD‐1 inhibitor. This study highlighted the clinical significance of VISTA and also offered possibilities to improve the immunotherapeutic strategy of ICB in gastric cancer.

## AUTHOR CONTRIBUTIONS

Yifan Cao was responsible for sample collection, experiments and data acquisition, analysis and interpretation, and drafting of the manuscript. Kuan Yu and Zihao Zhang were responsible for technical and material support, sample collection and western blot. Yun Gu was responsible for material support and contact with Dr. Jeeyun Lee (Department of Medicine, Samsung Medical Center, Sungkyunkwan University School of Medicine). Yichao Gu was responsible for material support. Weijuan Zhang and Wandi Li were responsible for flow cytometry analysis. Zhenbin Shen and Jiejie Xu were responsible for study concept and supervision. Jing Qin was responsible for study concept, design, supervision and obtaining funding. All the authors read and approved the final manuscript.

## CONFLICT OF INTEREST STATEMENT

The authors declare they have no conflicts of interest.

## ETHICS STATEMENT

All investigations were performed following the principles documented in the Declaration of Helsinki. The patients were informed of the usage of their biological samples. Written consent was acquired from each patient. The study was approved by the Clinical Research Ethics Committee of Zhongshan Hospital, Fudan University.

## Supporting information

Supporting Information

## Data Availability

Comprehensive patient characteristics of the TCGA and ACRG cohorts are publicly available in the TCGA database (http://cancergenome.nih.gov/) and GEO database (GSE62254). Patient characteristics of the SKKU‐SMC (PRJEB25780) cohort are available in TIDE (http://tide.dfci.harvard.edu/). Patient information of GSE134520 is available in GEO database or scTIME Portal (http://sctime.sklehabc.com/#/home). Patient characteristics of the PKU‐scDVA cohort are available at scDVA website (http://panmyeloid.cancer‐pku.cn/). The remaining data supporting the findings of this study are available within the article and supplementary files, or available from the authors upon request.

## References

[1] Nakamura Y , Shitara K , Lee J . The right treatment of the right patient: integrating genetic profiling into clinical decision making in advanced gastric cancer in Asia. Am Soc Clin Oncol Educ Book. 2021;41:e166‐e173. 10.1200/EDBK_321247 34010049

[2] Sung H , Ferlay J , Siegel RL , et al. Global Cancer Statistics 2020: GLOBOCAN estimates of incidence and mortality worldwide for 36 cancers in 185 countries. CA Cancer J Clin. 2021;71(3):209‐249. 33538338 10.3322/caac.21660

[3] Nakamura Y , Kawazoe A , Lordick F , et al. Biomarker‐targeted therapies for advanced‐stage gastric and gastro‐oesophageal junction cancers: an emerging paradigm. Nat Rev Clin Oncol. 2021;18(8):473‐487. 33790428 10.1038/s41571-021-00492-2

[4] Egen JG , Kuhns MS , Allison JP . CTLA‐4: new insights into its biological function and use in tumor immunotherapy. Nat Immunol. 2002;3(7):611‐618. 12087419 10.1038/ni0702-611

[5] Sharma P , Allison JP . The future of immune checkpoint therapy. Science. 2015;348(6230):56‐61. 25838373 10.1126/science.aaa8172

[6] Fuchs CS , Doi T , Jang RW , et al. Safety and efficacy of pembrolizumab monotherapy in patients with previously treated advanced gastric and gastroesophageal junction cancer: phase 2 clinical KEYNOTE‐059 trial. JAMA Oncol. 2018;4(5):e180013. 29543932 10.1001/jamaoncol.2018.0013 PMC5885175

[7] Janjigian YY , Bendell J , Calvo E , et al. CheckMate‐032 study: efficacy and safety of nivolumab and nivolumab plus ipilimumab in patients with metastatic esophagogastric cancer. J Clin Oncol. 2018;36(28):2836‐2844. 30110194 10.1200/JCO.2017.76.6212 PMC6161834

[8] Wang L , Rubinstein R , Lines JL , et al. VISTA, a novel mouse Ig superfamily ligand that negatively regulates T cell responses. J Exp Med. 2011;208(3):577‐592. 21383057 10.1084/jem.20100619 PMC3058578

[9] Lines JL , Sempere LF , Broughton T , et al. VISTA is a novel broad‐spectrum negative checkpoint regulator for cancer immunotherapy. Cancer Immunol Res. 2014;2(6):510‐517. 24894088 10.1158/2326-6066.CIR-14-0072 PMC4085258

[10] Le Mercier I , Chen W , Lines JL , et al. VISTA regulates the development of protective antitumor immunity. Cancer Res. 2014;74(7):1933‐1944. 24691994 10.1158/0008-5472.CAN-13-1506 PMC4116689

[11] Teft WA , Kirchhof MG , Madrenas J . A molecular perspective of CTLA‐4 function. Annu Rev Immunol. 2006;24(1):65‐97. 16551244 10.1146/annurev.immunol.24.021605.090535

[12] Hui E , Cheung J , Zhu J , et al. T cell costimulatory receptor CD28 is a primary target for PD‐1‐mediated inhibition. Science. 2017;355(6332):1428‐1433. 28280247 10.1126/science.aaf1292 PMC6286077

[13] Xu W , Hiếu T , Malarkannan S , et al. The structure, expression, and multifaceted role of immune‐checkpoint protein VISTA as a critical regulator of anti‐tumor immunity, autoimmunity, and inflammation. Cell Mol Immunol. 2018;15(5):438‐446. 29375120 10.1038/cmi.2017.148 PMC6068175

[14] Villarroel‐Espindola F , Yu X , Datar I , et al. Spatially resolved and quantitative analysis of VISTA/PD‐1H as a novel immunotherapy target in human non‐small cell lung cancer. Clin Cancer Res. 2018;24(7):1562‐1573. 29203588 10.1158/1078-0432.CCR-17-2542 PMC5884702

[15] Gao J , Ward JF , Pettaway CA , et al. VISTA is an inhibitory immune checkpoint that is increased after ipilimumab therapy in patients with prostate cancer. Nat Med. 2017;23(5):551‐555. 28346412 10.1038/nm.4308 PMC5466900

[16] Tagliamento M , Bironzo P , Novello S . New emerging targets in cancer immunotherapy: the role of VISTA. ESMO Open. 2020;4(suppl 3):e000683. 32554470 10.1136/esmoopen-2020-000683 PMC7305420

[17] Bass AJ , Thorsson V , Shmulevich I , et al. Comprehensive molecular characterization of gastric adenocarcinoma. Nature. 2014;513(7517):202‐209. 25079317 10.1038/nature13480 PMC4170219

[18] Cristescu R , Lee J , Nebozhyn M , et al. Molecular analysis of gastric cancer identifies subtypes associated with distinct clinical outcomes. Nat Med. 2015;21(5):449‐456. 25894828 10.1038/nm.3850

[19] Kim ST , Cristescu R , Bass AJ , et al. Comprehensive molecular characterization of clinical responses to PD‐1 inhibition in metastatic gastric cancer. Nat Med. 2018;24(9):1449‐1458. 30013197 10.1038/s41591-018-0101-z

[20] Zhang P , Yang M , Zhang Y , et al. Dissecting the single‐cell transcriptome network underlying gastric premalignant lesions and early gastric cancer. Cell Rep. 2019;27(6):1934‐1947.e5. 31067475 10.1016/j.celrep.2019.04.052

[21] Cheng S , Li Z , Gao R , et al. A pan‐cancer single‐cell transcriptional atlas of tumor infiltrating myeloid cells. Cell. 2021;184(3):792‐809.e23. 33545035 10.1016/j.cell.2021.01.010

[22] Cao Y , Liu H , Li H , et al. Association of O6‐methylguanine‐DNA methyltransferase protein expression with postoperative prognosis and adjuvant chemotherapeutic benefits among patients with stage II or III gastric cancer. JAMA Surg. 2017;152(11):e173120. 28903131 10.1001/jamasurg.2017.3120 PMC5831425

[23] Cao Y , He H , Li R , et al. Latency‐associated peptide identifies immunoevasive subtype gastric cancer with poor prognosis and inferior chemotherapeutic responsiveness. Ann Surg. 2022;275(1):e163‐e173. 32511132 10.1097/SLA.0000000000003833

[24] Picelli S , Faridani OR , Björklund ÅK , et al. Full‐length RNA‐seq from single cells using Smart‐seq2. Nat Protoc. 2014;9(1):171‐181. 24385147 10.1038/nprot.2014.006

[25] Huang DW , Sherman BT , Lempicki RA . Systematic and integrative analysis of large gene lists using DAVID bioinformatics resources. Nat Protoc. 2009;4(1):44‐57. 19131956 10.1038/nprot.2008.211

[26] El Tanbouly MA , Croteau W , Noelle RJ , et al. VISTA: a novel immunotherapy target for normalizing innate and adaptive immunity. Semin Immunol. 2019;42:101308. 31604531 10.1016/j.smim.2019.101308 PMC7233310

[27] Acharya N , Sabatos‐Peyton C , Anderson AC . Tim‐3 finds its place in the cancer immunotherapy landscape. J Immunother Cancer. 2020;8(1):e000911. 32601081 10.1136/jitc-2020-000911 PMC7326247

[28] Chauvin J‐M , Zarour HM . TIGIT in cancer immunotherapy. J Immunother Cancer. 2020;8(2):e000957. 32900861 10.1136/jitc-2020-000957 PMC7477968

[29] van Hall T , André P , Horowitz A , et al. Monalizumab: inhibiting the novel immune checkpoint NKG2A. J Immunother Cancer. 2019;7(1):263. 31623687 10.1186/s40425-019-0761-3 PMC6798508

[30] Doroshow DB , Bhalla S , Beasley MB , et al. PD‐L1 as a biomarker of response to immune‐checkpoint inhibitors. Nat Rev Clin Oncol. 2021;18(6):345‐362. 33580222 10.1038/s41571-021-00473-5

[31] Maruhashi T , Sugiura D , Okazaki I‐M , et al. LAG‐3: from molecular functions to clinical applications. J Immunother Cancer. 2020;8(2):e001014. 32929051 10.1136/jitc-2020-001014 PMC7488795

[32] Ishida Y , Agata Y , Shibahara K , et al. Induced expression of PD‐1, a novel member of the immunoglobulin gene superfamily, upon programmed cell death. EMBO J. 1992;11(11):3887‐3895. 1396582 10.1002/j.1460-2075.1992.tb05481.x PMC556898

[33] Böger C , Behrens H‐M , Krüger S , et al. The novel negative checkpoint regulator VISTA is expressed in gastric carcinoma and associated with PD‐L1/PD‐1: a future perspective for a combined gastric cancer therapy? OncoImmunology. 2017;6(4):e1293215. 28507801 10.1080/2162402X.2017.1293215 PMC5414883

[34] Schlichtner S , Yasinska IM , Lall GS , et al. T lymphocytes induce human cancer cells derived from solid malignant tumors to secrete galectin‐9 which facilitates immunosuppression in cooperation with other immune checkpoint proteins. J Immunother Cancer. 2023;11(1):e005714. 36599470 10.1136/jitc-2022-005714 PMC9815087

[35] Schlichtner S , Yasinska IM , Ruggiero S , et al. Expression of the immune checkpoint protein VISTA is differentially regulated by the TGF‐β1–Smad3 signaling pathway in rapidly proliferating human cells and T lymphocytes. Front Med. 2022;9:790995. 10.3389/fmed.2022.790995 PMC8866318 35223897

[36] Yasinska IM , Meyer NH , Schlichtner S , et al. Ligand–receptor interactions of galectin‐9 and VISTA suppress human T lymphocyte cytotoxic activity. Front Immunol. 2020;11:580557. 33329552 10.3389/fimmu.2020.580557 PMC7715031

[37] Zhang Q , He Y , Luo N , et al. Landscape and dynamics of single immune cells in hepatocellular carcinoma. Cell. 2019;179(4):829‐845.e20. 31675496 10.1016/j.cell.2019.10.003

[38] Zhang L , Li Z , Skrzypczynska KM , et al. Single‐cell analyses inform mechanisms of myeloid‐targeted therapies in colon cancer. Cell. 2020;181(2):442‐459.e29. 32302573 10.1016/j.cell.2020.03.048

[39] Palani S , Maksimow M , Miiluniemi M , et al. Stabilin‐1/CLEVER‐1, a type 2 macrophage marker, is an adhesion and scavenging molecule on human placental macrophages. Eur J Immunol. 2011;41(7):2052‐2063. 21480214 10.1002/eji.201041376

[40] Timperi E , Gueguen P , Molgora M , et al. Lipid‐associated macrophages are induced by cancer‐associated fibroblasts and mediate immune suppression in breast cancer. Cancer Res. 2022;82(18):3291‐3306. 35862581 10.1158/0008-5472.CAN-22-1427

[41] Martinez FO , Gordon S . The M1 and M2 paradigm of macrophage activation: time for reassessment. F1000Prime Rep. 2014;6:13. 24669294 10.12703/P6-13 PMC3944738

[42] Xuan W , Qu Q , Zheng B , et al. The chemotaxis of M1 and M2 macrophages is regulated by different chemokines. J Leukocyte Biol. 2014;97(1):61‐69. 25359998 10.1189/jlb.1A0314-170R

[43] dos Santos AG , Mendes ÉA , de Oliveira RP , et al. Trichoderma asperelloides spores downregulate dectin1/2 and TLR2 receptors of mice macrophages and decrease candida parapsilosis phagocytosis independent of the M1/M2 polarization. Front Microbiol. 2017;8:1681. 28936201 10.3389/fmicb.2017.01681 PMC5594820

[44] Im JH , Yeo IJ , Park PH , et al. Deletion of Chitinase‐3‐like 1 accelerates stroke development through enhancement of neuroinflammation by STAT6‐dependent M2 microglial inactivation in Chitinase‐3‐like 1 knockout mice. Exp Neurol. 2020;323:113082. 31669069 10.1016/j.expneurol.2019.113082

[45] Chen X , Gao Y , Xie J , et al. Identification of FCN1 as a novel macrophage infiltration‐associated biomarker for diagnosis of pediatric inflammatory bowel diseases. J Transl Med. 2023;21(1):203. 36932401 10.1186/s12967-023-04038-1 PMC10022188

[46] Jouvene CC , Shay AE , Soens MA , et al. Biosynthetic metabolomes of cysteinyl‐containing immunoresolvents. FASEB J. 2019;33(12):13794‐13807. 31589826 10.1096/fj.201902003R PMC6894058

[47] Wang H , Wang Q , Wu Y , et al. Autophagy‐related gene LAPTM4B promotes the progression of renal clear cell carcinoma and is associated with immunity. Front Pharmacol. 2023;14:1118217. 36937841 10.3389/fphar.2023.1118217 PMC10017457

[48] Wang Y , Yan K , Lin J , et al. Macrophage M2 co‐expression factors correlate with the immune microenvironment and predict outcome of renal clear cell carcinoma. Front Genet. 2021;12:615655. 33692827 10.3389/fgene.2021.615655 PMC7938896

[49] Wu J , Liu X , Wu J , et al. CXCL12 derived from CD248‐expressing cancer‐associated fibroblasts mediates M2‐polarized macrophages to promote nonsmall cell lung cancer progression. Biochim Biophys Acta Mol Basis Dis. 2022;1868(11):166521. 35985448 10.1016/j.bbadis.2022.166521

[50] Noubade R , Wong K , Ota N , et al. NRROS negatively regulates reactive oxygen species during host defence and autoimmunity. Nature. 2014;509(7499):235‐239. 24739962 10.1038/nature13152

[51] Liu J , Yuan Y , Chen W , et al. Immune‐checkpoint proteins VISTA and PD‐1 nonredundantly regulate murine T‐cell responses. Proc Natl Acad Sci U S A. 2015;112(21):6682‐6687. 25964334 10.1073/pnas.1420370112 PMC4450438

[52] Zhang M , Zhang J , Liu N , et al. VISTA is associated with immune infiltration and predicts favorable prognosis in TNBC. Front Oncol. 2022;12:961374. 36158663 10.3389/fonc.2022.961374 PMC9493462

[53] Cao X , Ren X , Zhou Y , et al. VISTA expression on immune cells correlates with favorable prognosis in patients with triple‐negative breast cancer. Front Oncol. 2021;10:583966. 33505908 10.3389/fonc.2020.583966 PMC7829913

[54] Loeser H , Kraemer M , Gebauer F , et al. The expression of the immune checkpoint regulator VISTA correlates with improved overall survival in pT1/2 tumor stages in esophageal adenocarcinoma. OncoImmunology. 2019;8(5):e1581546. 31069143 10.1080/2162402X.2019.1581546 PMC6492979

[55] Kuklinski LF , Yan S , Li Z , et al. VISTA expression on tumor‐infiltrating inflammatory cells in primary cutaneous melanoma correlates with poor disease‐specific survival. Cancer Immunol Immunother. 2018;67(7):1113‐1121. 29737375 10.1007/s00262-018-2169-1 PMC11028124

[56] Lines JL , Pantazi E , Mak J , et al. VISTA is an immune checkpoint molecule for human T cells. Cancer Res. 2014;74(7):1924‐1932. 24691993 10.1158/0008-5472.CAN-13-1504 PMC3979527

[57] Xie X , Chen C , Chen W , et al. Structural basis of VSIG3: the ligand for VISTA. Front Immunol. 2021;12:625808. 33841409 10.3389/fimmu.2021.625808 PMC8027081

[58] Schaafsma E , Croteau W , El Tanbouly M , et al. VISTA targeting of T‐cell quiescence and myeloid suppression overcomes adaptive resistance. Cancer Immunol Res. 2023;11(1):38‐55. 36260656 10.1158/2326-6066.CIR-22-0116 PMC10544831

