# Supplementary material for: Blockade of V‐domain immunoglobulin suppressor of T‐cell activation reprograms tumour‐associated macrophages and improves efficacy of PD‐1 inhibitor in gastric cancer
Source: Clin Transl Med. 2024 Feb 15;14(2):e1578. doi: 10.1002/ctm2.1578 (PMC10867598; doi:10.1002/ctm2.1578)
Supplement: Supplementary file 1 — Supporting Information [file CTM2-14-e1578-s001.docx]

**Blockade of V-domain immunoglobulin suppressor of T cell activation reprograms tumor-associated macrophages and improves efficacy of PD-1 inhibitor in gastric cancer**

**Running title:** **VISTA identifies immunoevasive gastric cancer**

Yifan Cao^1^, M.D., Ph.D.; Kuan Yu^1^, M.D., Ph.D.; Zihao Zhang^1^, M.D., Ph.D.; Yun Gu^1^, M.D.; Yichao Gu^1^, M.D., Ph.D.; Wandi Li^2^, Ph.D.; Weijuan Zhang^2^, M.D., Ph.D.; Zhenbin Shen^1,*^, M.D.; Jiejie Xu^3,*^, M.D., Ph.D.; Jing Qin^1,*^, M.D.

^1^Department of General Surgery, Zhongshan Hospital, Fudan University, Shanghai, China;

^2^Department of Immunology, School of Basic Medical Sciences, Fudan University, Shanghai, China;

^3^Department of Biochemistry and Molecular Biology, School of Basic Medical Sciences, Fudan University, Shanghai, China.

****Corresponding authors.*** Department of General Surgery, Zhongshan Hospital, Fudan University, Shanghai, China. E-mail address: [shen.zhenbin@zs-hospital.sh.cn](mailto:shen.zhenbin@zs-hospital.sh.cn) **(Z. Shen)**; Department of Biochemistry and Molecular Biology, School of Basic Medical Sciences, Fudan University, Shanghai, China. E-mail address: [jjxufdu@fudan.edu.cn](mailto:jjxufdu@fudan.edu.cn) (**J. Xu**); Department of General Surgery, Zhongshan Hospital, Fudan University, Shanghai, China. E-mail address: [qin.jing@zs-hospital.sh.cn](mailto:qin.jing@zs-hospital.sh.cn) **(J. Qin)**

Supplementary Method S1. Construction of TMA.

Supplementary Method S2. Analysis of VISTA expression in gastric cancer.

Supplementary Figure S1. Patient composition of the study.

Supplementary Figure S2. Comparison of VISTA expression in tumor tissues and peritumor tissues.

Supplementary Figure S3. Scoring system for VISTA protein expression in gastric cancer.

Supplementary Figure S4. Inter-observer agreement in the evaluation of VISTA protein expression.

Supplementary Figure S5. Comparison between predictive power of significant clinical parameters in gastric cancer.

Supplementary Figure S6. Single cell RNA sequencing analysis explores expression of *VSIR* in gastric cancer.

Supplementary Figure S7. Gating strategies for flow cytometry.

Supplementary Figure S8. qPCR and WB validate the expression of VISTA in TAMs.

Supplementary Figure S9. VISTA^+^ TAM signature predicts survival outcomes in gastric cancer.

Supplementary Figure S10. Gating strategies to detect the effect of VISTA blockade on phenotype of TAMs in gastric cancer.

Supplementary Table S1. List of gastric cancer datasets.

Supplementary Table S2. List of antibodies used in the study.

Supplementary Table S3. List of software and algorithms used in the study.

Supplementary Table S4. Real-time quantitative PCR primers.

Supplementary Table S5. List of genes selected for VISTA^+^ TAM signature.

**Supplementary Method S1. Construction of TMA.**

Formalin-fixed and paraffin-embedded (FFPE) gastric tumor tissue and/or peritumor tissue blocks were retrospectively acquired from gastric cancer patients who underwent radical gastrectomy and standard D2 lymphadenectomy between August 2007 and December 2008 (T13-564)^1^ or between August 2018 and November 2018 (T19-0097)^2^ in Zhongshan Hospital Fudan University (FDU-ZSH). Tissue microarray (TMA) slides were constructed with the help of Shanghai Outdo Biotech Co., Ltd (Shanghai, China). Briefly, testing sections were sliced from each FFPE tissue block, and performed with hematoxylin and eosin (H&E) staining for selection and confirmation of representative core area. Subsequently, 2-mm-wide tissue core cylinders were punched from the marked core area of each tissue block, and transferred to a recipient block, which was called as TMA block. Then, the TMA blocks were consecutively sliced into 4-μm-thick sections and paved on microscope slides. The TMA slides (T13-564 and T19-0097) were ultimately constructed. One of the sections from each TMA block was performed with H&E staining to ensure that the TMA slides were adequately constructed.

**Supplementary Method S2. Analysis of VISTA expression in gastric cancer.**

Expression of VISTA protein was scored in a blinded fashion. A detailed description of the IHC scoring system, together with representative images and scoring results, was provided **(Supplementary Figure S2-4)**. Briefly, two independent investigators scored VISTA expression under the same magnification (×200). In gastric cancer, tumor tissues showed higher expression of VISTA than peritumor tissues **(Supplementary Figure S2)**. Moreover, VISTA was predominantly expressed in tumor stroma, whereas malignant epithelial showed almost no expression of VISTA **(Supplementary Figure S3)**. Consequently, VISTA expression was evaluated as the average number of VISTA^+^ cells from 3 randomized fields (magnification: ×200). The IHC staining intensity of VISTA was stratified as 0 (no staining), 1 (weak staining, 0<[VISTA^+^cells]≤5), 2 (moderate staining, 5<[VISTA^+^cells]≤20), and 3 (strong staining, [VISTA^+^cells]>20; **Supplementary Figure S3**). To assess the robustness of our scoring system, and avoid possible bias introduced by inter-observer variability, we analyzed the concordance between the scoring results given from two independent investigators with the use of contingency tables and calculation of Cohen’s Kappa Indices **(Supplementary Figure S4)**. The original IHC scores from the two independent investigators were in good concordance (κ = 0.90, 95% confidence interval [CI]: 0.86-0.93; **Supplementary Figure S4A**). Notably, there was a significant improvement in concordance (κ = 0.98, 95% CI: 0.96-1.00), if we classified Score 0 and Score 1 as VISTA^low^, while Score 2 and Score 3 as VISTA^high^ **(Supplementary Figure S4B)**. Hence, we randomly chose the results from Observer #2 as the standard VISTA status. Finally, the association between VISTA expression and survival outcomes was tested by a third investigator, who did not participate in the scoring process.


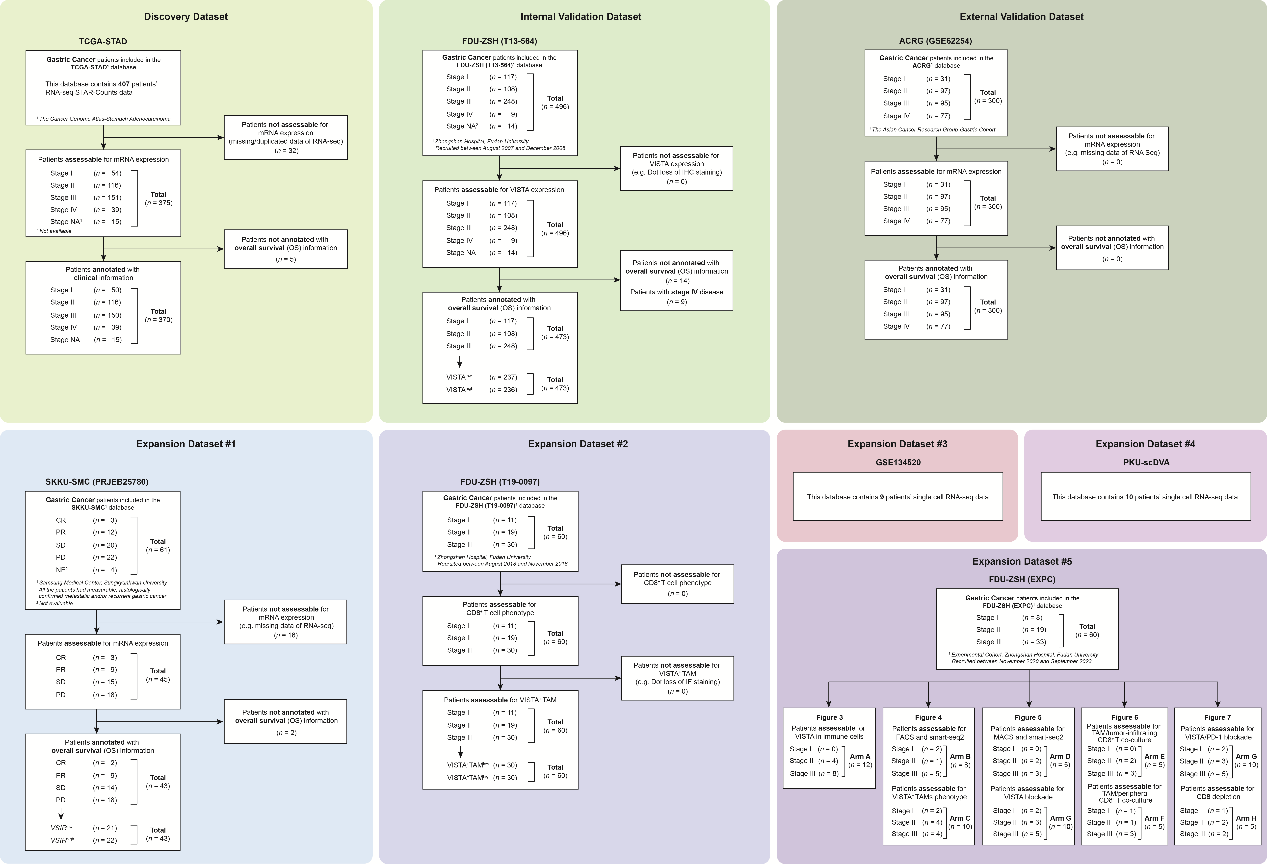


**Supplementary Figure S1. Patient composition of the study.** Our study enrolled 8 independent datasets with a total of 1,403 gastric cancer patients. Discovery Dataset (TCGA-STAD, *n* = 407),^3^ External Validation Dataset (GSE62254 ACRG, *n* = 300),^4^ Expansion Dataset #1 (SKKU-SMC, *n* = 61),^5^ Expansion Dataset #3 (GSE134520, *n* = 9)^6^ and Expansion Dataset #4 (PKU-scDVA, *n* = 10)^7^ were public datasets. Internal Validation Dataset (T13-564, *n* = 496),^1^ Expansion Dataset #2 (T19-0097, *n* = 60)^2^ and Expansion Dataset #5 (EXPC, *n* = 60) were our own datasets and were recruited from FDU-ZSH. All the patients recruited from FDU-ZSH did not have any treatment before surgery. The SKKU-SMC immunotherapy cohort was derived from a prospective, open-label, single-arm, phase 2 trial conducted at Samsung Medical Center, Sungkyunkwan University.^5^ Pembrolizumab 200 mg was administered every 3 weeks until documented disease progression, unacceptable toxicity, or up to 24 months. Tumor responses were evaluated every two cycles according to RECIST 1.1 criteria. Tumor tissues were obtained before initiation of immunotherapy. Abbreviations: TCGA-STAD, the Cancer Genome Atlas-Stomach Adenocarcinoma; ACRG, the Asian Cancer Research Group; SKKU-SMC, Samsung Medical Center, Sungkyunkwan University; EXPC, Experimental Cohort; FDU-ZSH, Zhongshan Hospital, Fudan University; PKU-scDVA, Peking University-short for single cell RNA-seq data visualization and analyzation.


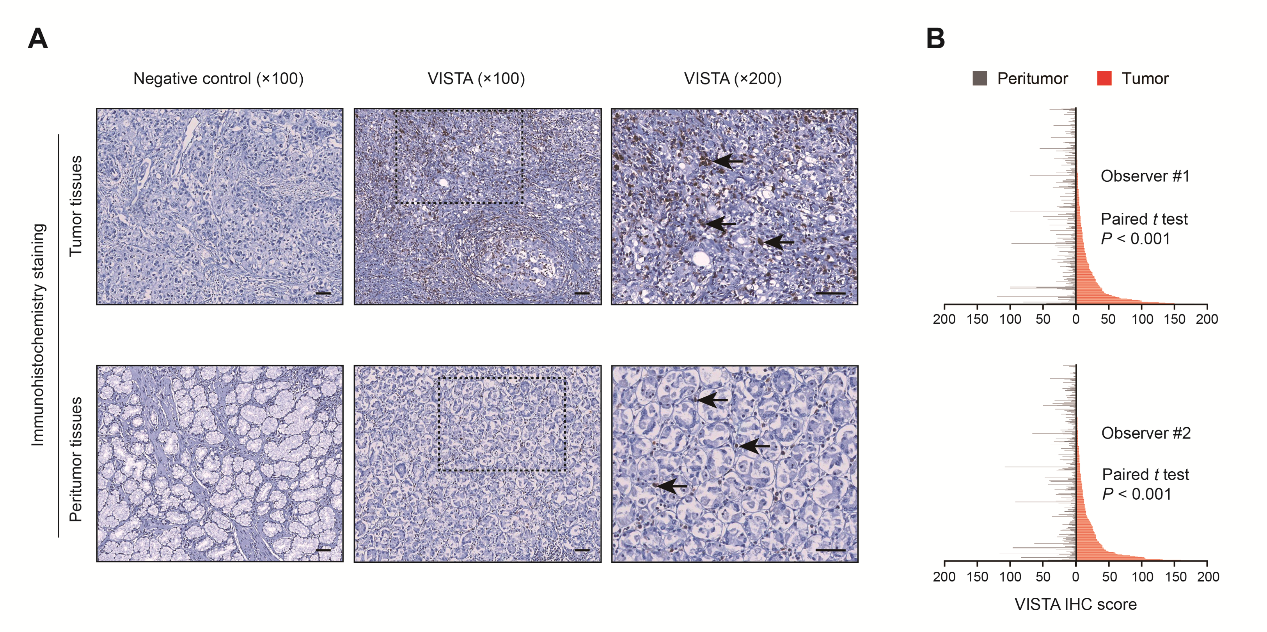


**Supplementary Figure S2. Comparison of VISTA expression in tumor tissues and peritumor tissues. (A)** TMAs of FDU-ZSH (T13-564) were performed with immunohistochemistry (IHC) to detect VISTA expression in gastric cancer tissues and peritumor tissues. **(B)** According to the IHC score given by the two independent observers, both observers found that tumor tissues had significantly higher expression of VISTA than peritumor tissues. Significance was determined by paired *t* test. Arrows show VISTA^+^ cells. Scale bar represents 50 μm.


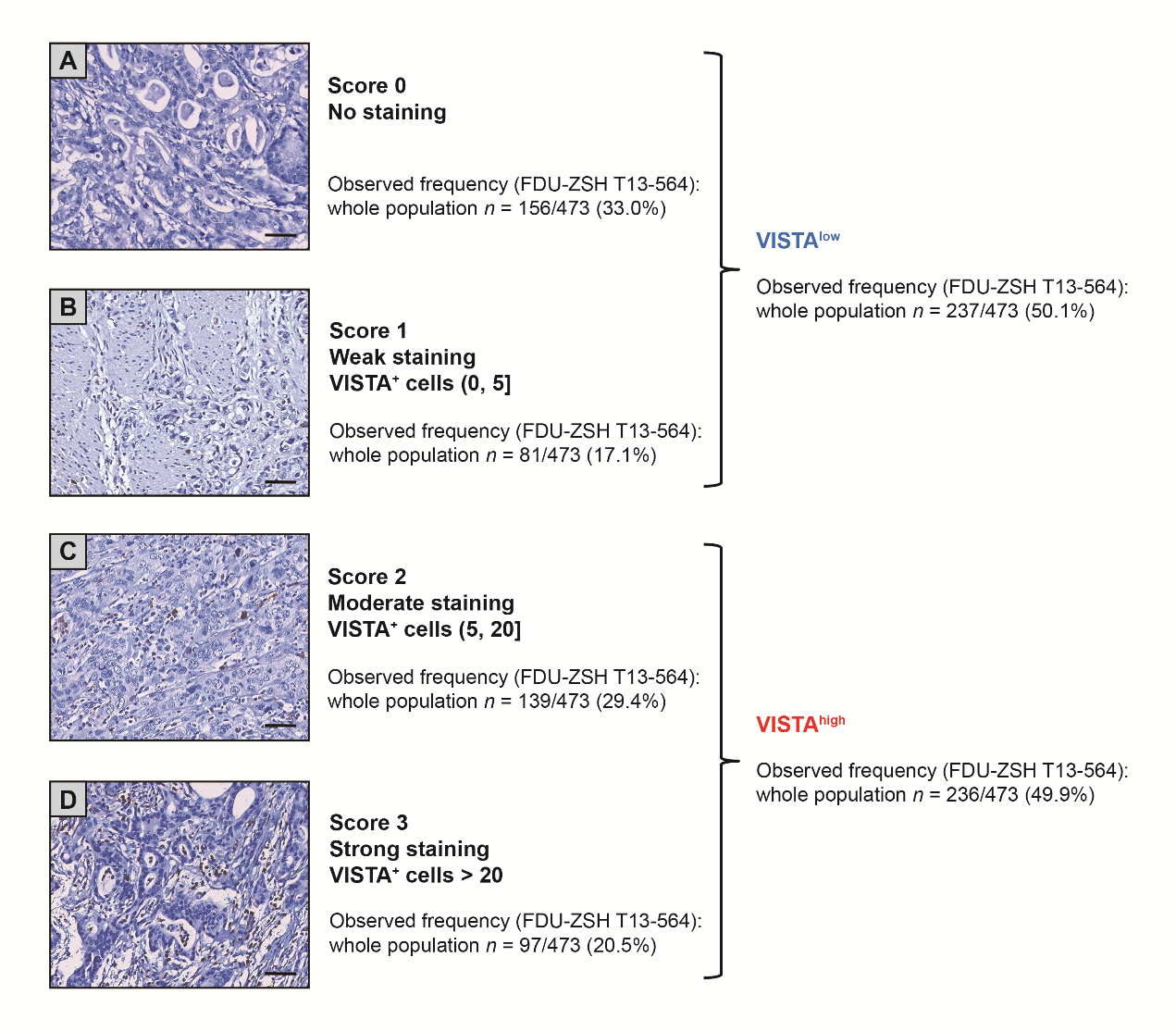


**Supplementary Figure S3. Scoring system for VISTA protein expression in gastric cancer.** Patients with gastric cancer were stratified into two subgroups: VISTA^low^ subgroup and VISTA^high^ subgroup. The two observers scored the tumors in which VISTA^+^ cells ≤ 5 as VISTA^low^. Tumors scored as VISTA^low^ had two staining patterns: **(A)** complete lack of VISTA expression (Score 0); **(B)** weak staining of VISTA (Score 1; 0 < VISTA^+^ cells ≤ 5). The two observers scored the tumors in which VISTA^+^ cells > 5 as VISTA^high^. Tumors scored as VISTA^high^ also had two staining patterns: **(C)** moderate staining of VISTA (Score 2; 5 < VISTA^+^ cells ≤ 20); **(D)** strong staining of VISTA (Score 3; VISTA^+^ cells > 20). Scale bar represents 50 μm.


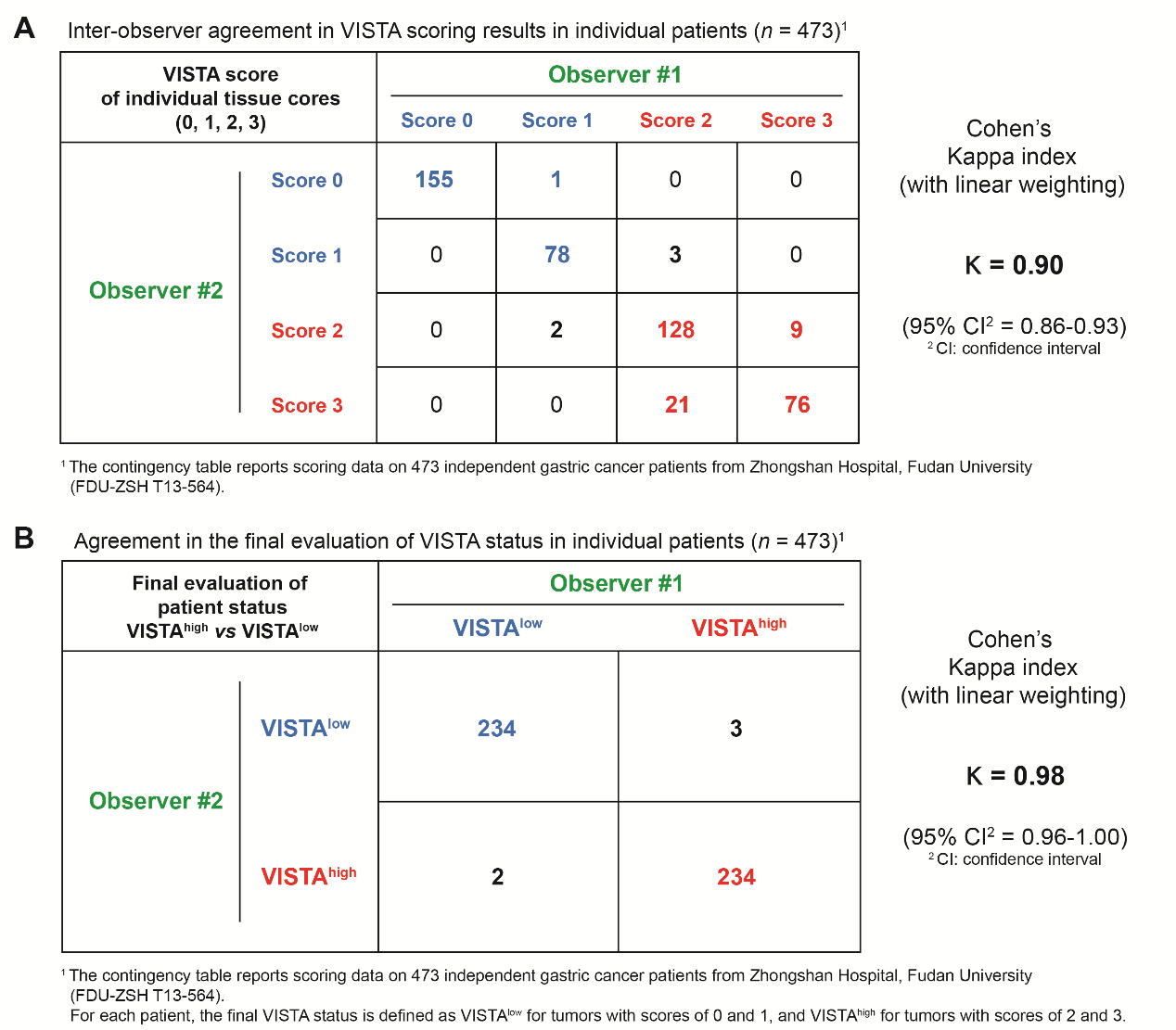


**Supplementary Figure S4. Inter-observer agreement in the evaluation of VISTA protein expression.** Two independent observers used the same criteria **(Supplementary Figure S3)** to independently score VISTA expression. The concordance between the two observers was analyzed using contingency tables to calculate the Cohen’s Kappa Index. The results showed an agreement (κ ≥ 0.9), both in terms of VISTA scoring of the individual patients **(A)** and in the final VISTA status of individual patients **(B)**. Most importantly, these results showed a good agreement (κ = 0.98) with regard to the final assessment of the patients’ VISTA status (B). We randomly selected the VISTA score given by Observer #2 as the standard VISTA score in this study.


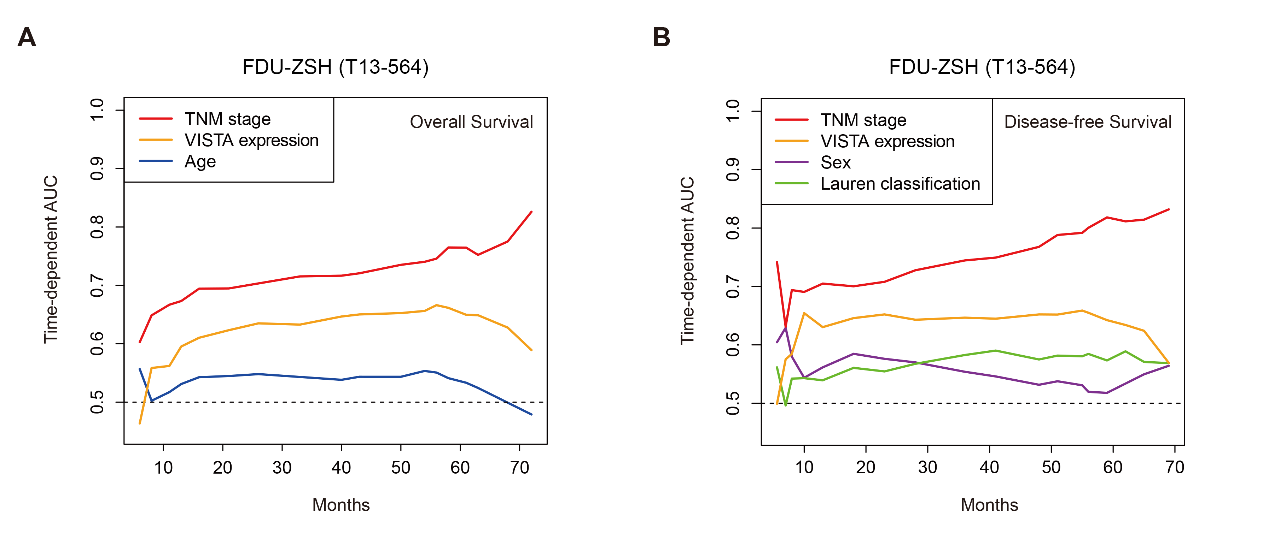
**Supplementary Figure S5. Comparison between predictive power of significant clinical parameters in gastric cancer. (A)** Time-dependent AUC (TDAUC) was conducted in FDU-ZSH (T13-564) to investigate the predictive power of TNM stage, VISTA expression and age on overall survival. **(B)** Time-dependent AUC was conducted in FDU-ZSH (T13-564) to investigate the predictive power of TNM stage, VISTA expression, sex and Lauren classification on disease-free survival.


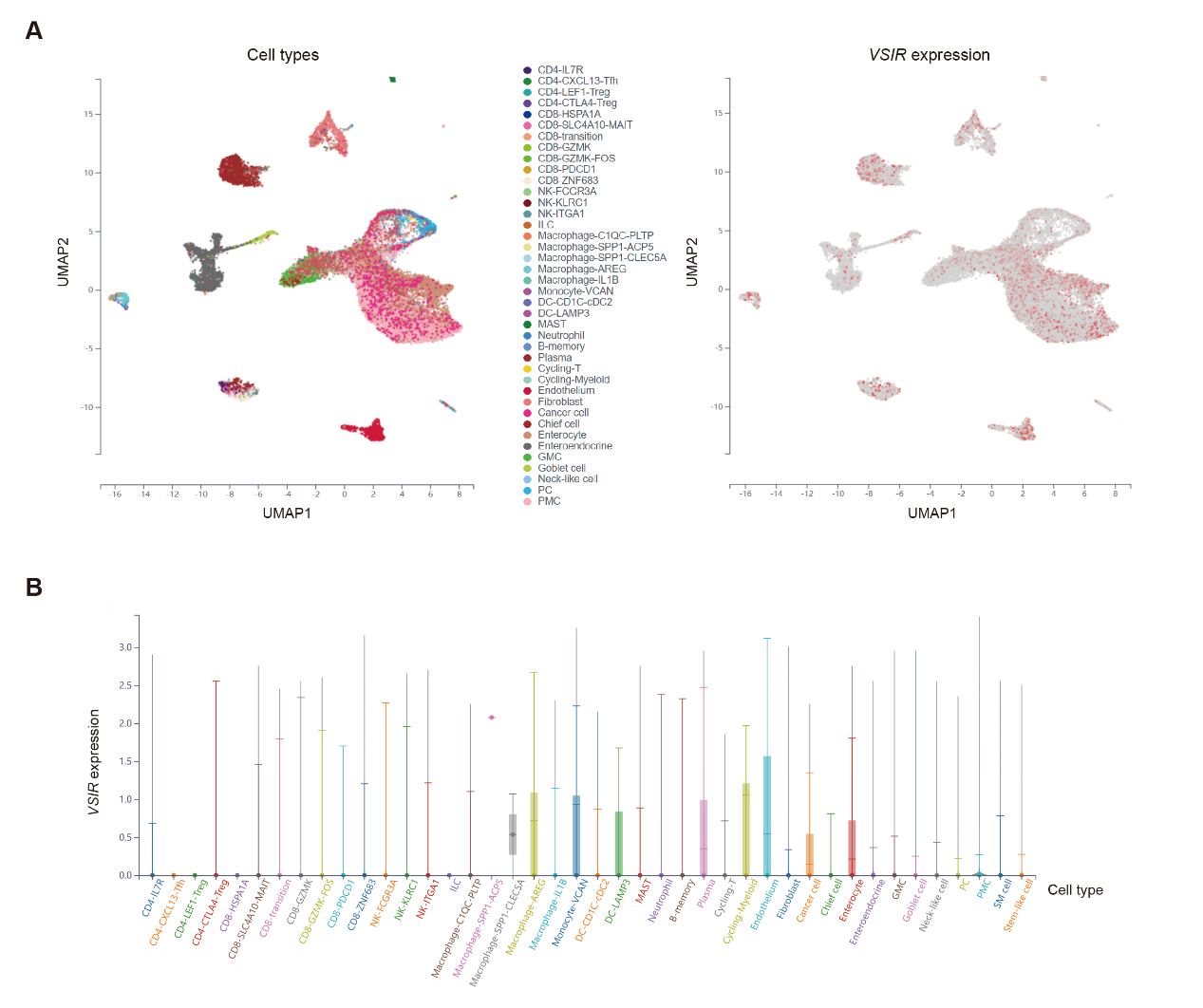
**Supplementary Figure S6.** **Single cell RNA sequencing analysis explores expression of *VSIR* in gastric cancer.** According to Expansion Dataset #3 (GSE134520), *VSIR* was potentially expressed on several kinds of cells, including monocytes/macrophages and dendritic cells (DCs) in gastric cancer. GSE134520 dataset contains 9 patients and is available through scTIME Portal (<http://sctime.sklehabc.com/#/home>).


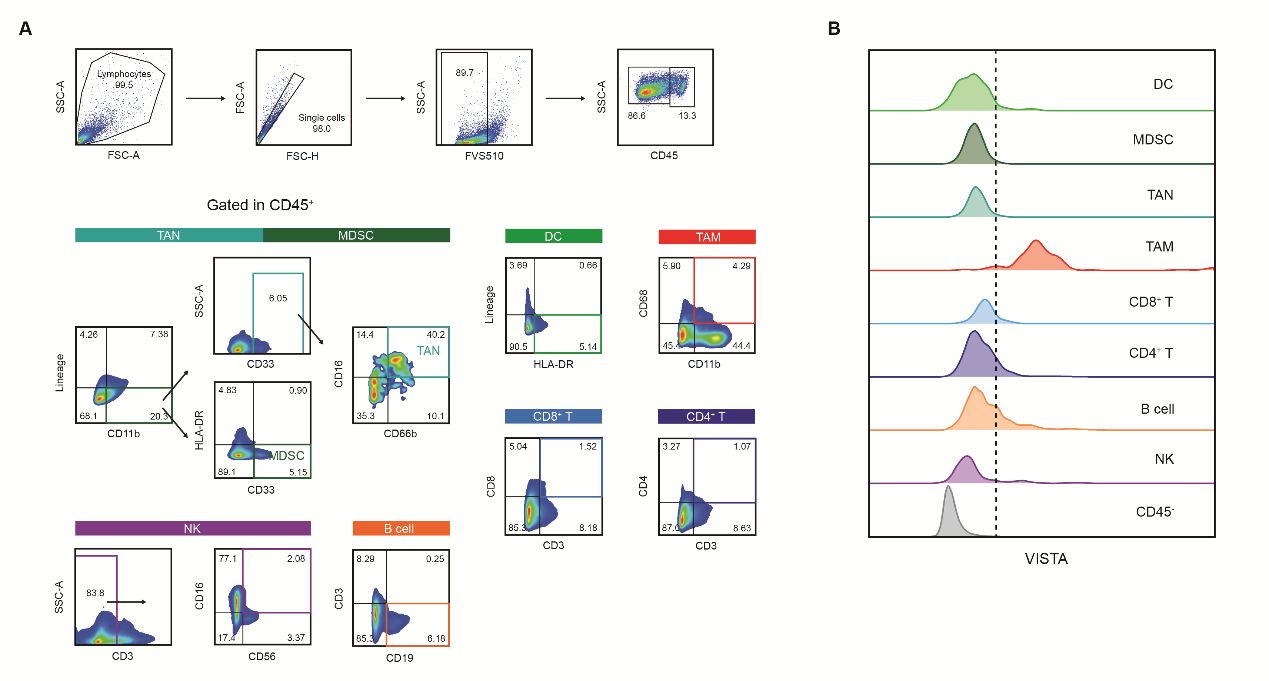


**Supplementary Figure S7. Gating strategies for flow cytometry.** We collected 12 fresh gastric cancer samples from FDU-ZSH EXPC (Arm A), and performed FC to validate the distribution of VISTA. Gating strategies for immune cells **(A)**, and percentage of VISTA^+^ cells in each cell type **(B)** was shown, respectively. DC, dendritic cell; MDSC, myeloid-derived suppressor cell; TAN, tumor-associated neutrophil; TAM, tumor-associated macrophage; NK, natural killer cell.


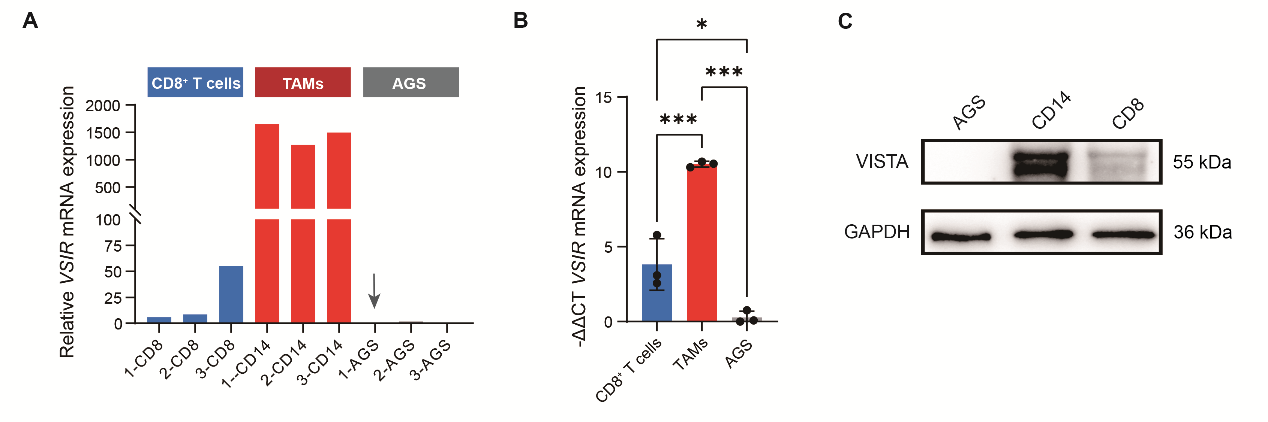


**Supplementary Figure S8. qPCR and WB validate the expression of VISTA in TAMs. (A-B)** Three fresh gastric cancer samples from FDU-ZSH EXPC (Arm A) were selected to isolate tumor-infiltrating CD8^+^ T cells and tumor-associated macrophages (TAMs) by means of MACS. qPCR validated that TAMs showed higher *VSIR* mRNA level than CD8^+^ T cells or AGS cells. **(C)** According to WB, TAMs showed significantly higher expression of VISTA compared with CD8^+^ T cells or AGS cells. Additionally, we found that TAMs expressed 55 kDa VISTA, which meant VISTA was expressed by TAMs themselves other than an extracellular domain proteolytically shed off the surface of other cells. Arrow shows the AGS sample #1 which was designated as reference for *VSIR* mRNA relative quantity. Significance values were determined by one-way ANOVA followed by Tukey’s multiple comparisons test (B). ****P* < 0.001, **P* < 0.05.


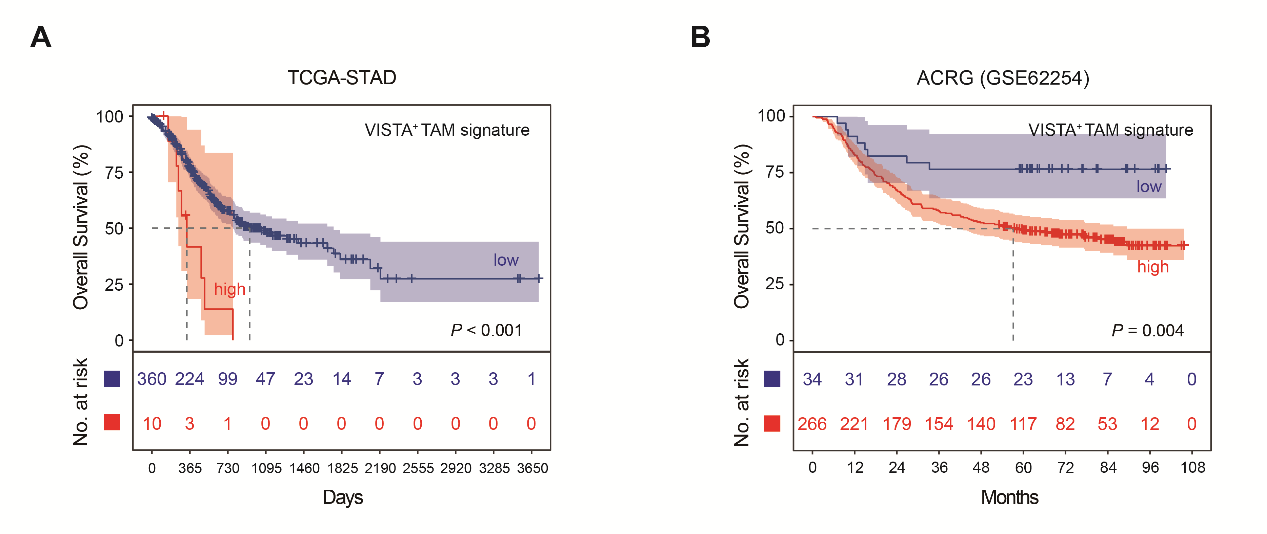


**Supplementary Figure S9. VISTA^+^ TAM signature predicts survival outcomes in gastric cancer.** According to the differentially expressed genes given by smart-seq2 **(Supplementary Table S5)**, we constructed a VISTA^+^ TAM-specific signature. The patients with high VISTA^+^ TAM signature had significantly poorer overall survival in TCGA-STAD dataset **(A)** and ACRG (GSE62254) dataset **(B)**. Significance was determined by Log-rank test. Cut-off value was defined by Cutoff Finder (<http://molpath.charite.de/cutoff>).^8^


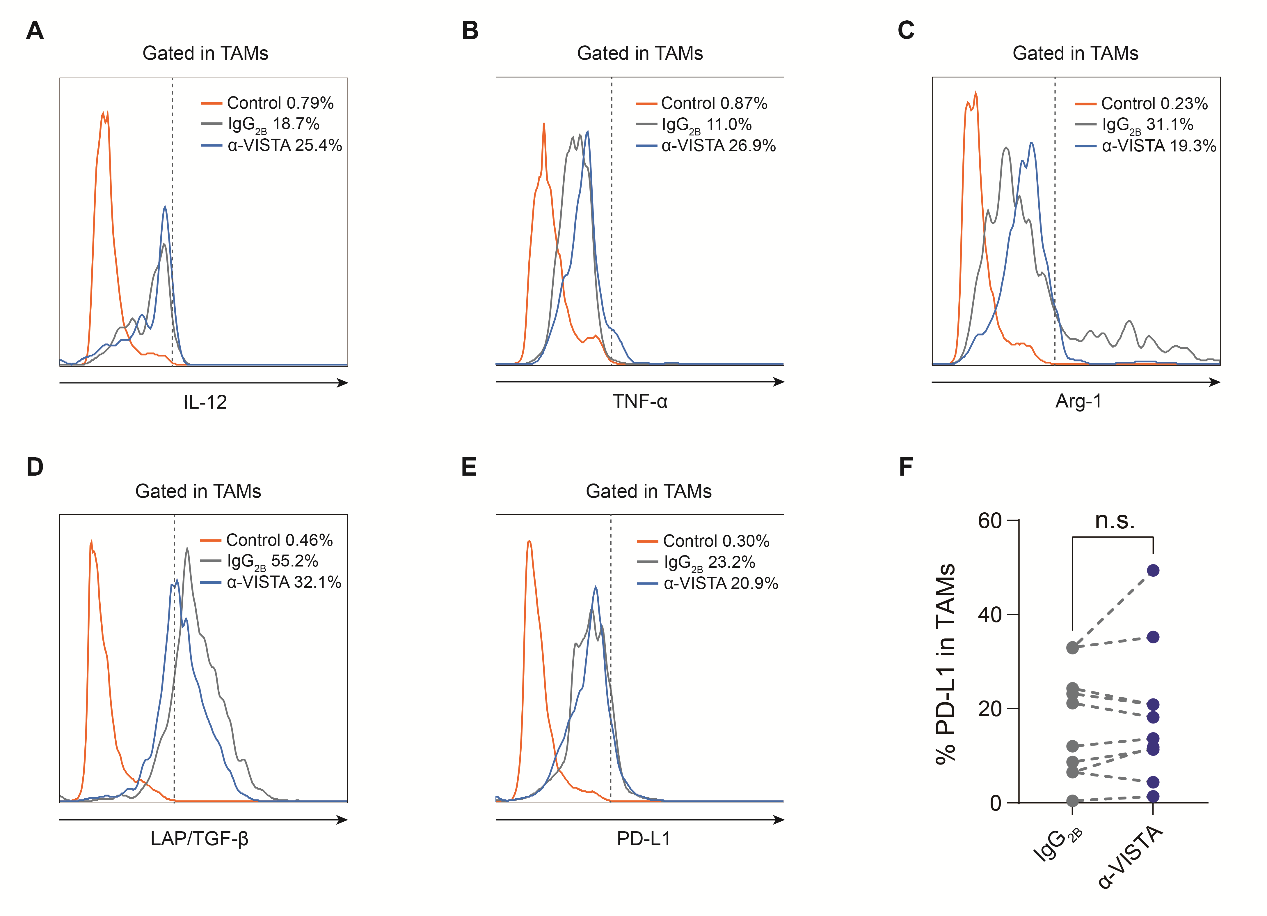
**Supplementary Figure S10. Gating strategies to detect the effect of VISTA blockade on phenotype of TAMs in gastric cancer.** Fresh gastric cancer specimens were collected and incubated with anti-VISTA (α-VISTA) antibody or isotype control (IgG_2B_), and then performed with FC/ICFC to detect the expression of IL-12 **(A)**, TNF-α **(B)**, Arg-1 **(C)**, LAP/TGF-β **(D)** and PD-L1 **(E-F)** within TAMs (FDU-ZSH EXPC Arm G). Significance value was determined by Wilcoxon matched-pairs signed rank test (F). n.s. refers to not significant. Abbreviations: Arg-1, arginase-1; TGF-β, transforming growth factor-β; PD-L1, programmed cell death 1-ligand 1; IL-12, interleukin-12; TNF-α, tumor necrosis factor-α.

| **Supplementary Table S1. List of gastric cancer datasets.** | | | |
| --- | --- | --- | --- |
| **Gastric cancer datasets** | **Number of patients** | **PubMed ID** | **Reference** |
| **Publicly available Gastric Cancer Datasets** | **787** |  |  |
| TCGA-STAD^1^ | 407 | PMID 25079317 | TCGA. *Nature*. 2014;513(7517):202-9 |
| ACRG^2^ (GSE62254) | 300 | PMID 25894828 | Cristescu R, et al. *Nat Med*. 2015;21(5):449-56 |
| SKKU-SMC^3^ (PRJEB25780) | 61 | PMID 30013197 | Kim ST, et al. *Nat Med*. 2018;24(9):1449-1458 |
| PKU-scDVA^4^ | 10 | PMID: 33545035 | Cheng S, et al. *Cell*. 2021;184(3):792-809.e23. |
| GSE134520 | 9 | PMID 31067475 | Zhang P, et al. *Cell Rep*. 2019 May 7;27(6):1934-1947.e5 |
| **FDU-ZSH^5^ Gastric Cancer Datasets** | **616** |  |  |
| T13-564 | 496 | PMID 28903131 | Cao Y, et al. *JAMA Surg*. 2017;152(11):e173120 |
| T19-0097 | 60 | PMID 32511132 | Cao Y, et al. *Ann Surg*. 2022;275(1):e163-e173 |
| EXPC^6^ | 60 | This paper | This paper |
| ^1^The Cancer Genome Atlas-Stomach Adenocarcinoma. (<http://cancergenome.nih.gov/>);  ^2^Asian Cancer Research Group. (<http://www.asiancancerresearchgroup.org/>);  ^3^Samsung Medical Center, Sungkyunkwan University;  ^4^Peking University-short for single cell RNA seq data visualization and analyzation. (<http://panmyeloid.cancer-pku.cn/>);  ^5^Zhongshan Hospital, Fudan University. T13-564, T19-0097 and EXPC were our own patient cohorts;  ^6^Experimental Cohort. | | | |

| **Supplementary Table S2. List of antibodies used in the study.** | | | |
| --- | --- | --- | --- |
| **Product** | **Manufacturer** | **Identifier** | **Application** |
| Anti-Human VISTA Antibody [BLR035F] | Abcam | Cat# ab243891 | IHC^1^, IF^2^ |
| Anti-Human PD-L1 Antibody [SP142] | Abcam | Cat# ab228462 | IHC |
| Anti-Human CD68 Antibody [KP1] | Abcam | Cat# ab955 | IHC, IF |
| Anti-Human CD8 Antibody | Dako | Clone C8/144B | IHC |
| Anti-Human MLH1 antibody | Zsbio | Cat# ZM-0154 | IHC |
| Human BD Fc Block NALE | BD Biosciences | Cat#564765 | FC^3^ |
| Fixable Viability Stain 510 | BD Biosciences | Cat# 564406 | FC |
| PE/Cyanine7 Anti-Human CD45 Antibody | BioLegend | Cat# 368532 | FC |
| FITC Anti-Human CD8 Antibody | BD Biosciences | Cat# 555366 | FC |
| PE Anti-Human CD279 (PD-1) Antibody | BD Biosciences | Cat# 560795 | FC |
| BV605 Anti-Human CD152 (CTLA-4) Antibody | BioLegend | Cat# 369610 | FC |
| AF647 Anti-Human TIM-3 (CD366) Antibody | BD Biosciences | Cat# 565558 | FC |
| BV785 Anti-Human CD223 (LAG-3) Antibody | BioLegend | Cat# 369322 | FC |
| APC-R700 Anti-Human IFN-γ Antibody | BD Biosciences | Cat# 564981 | ICFC^4^ |
| PE Anti-Human Granzyme B Antibody | Thermo Fisher Scientific | Cat# MA5-23688 | ICFC |
| AF647 Anti-Human Perforin Antibody | BD Biosciences | Cat# 563576 | ICFC |
| APC/Cyanine7 Anti-Human CD45 Antibody | BioLegend | Cat# 368516 | FC |
| PerCP/Cyanine5.5 Anti-Human CD68 Antibody | BioLegend | Cat# 333814 | ICFC |
| BV421 Anti-Human CD66 Antibody | BD Biosciences | Cat# 562741 | FC |
| BV605 Anti-Human CD4 Antibody | BioLegend | Cat# 300556 | FC |
| FITC Anti-Human CD8 Antibody | BioLegend | Cat# 344704 | FC |
| PE/Cyanine7 Anti-Human CD11c Antibody | BioLegend | Cat# 301608 | FC |
| BV785 Anti-Human HLA-DR Antibody | BioLegend | Cat# 307642 | FC |
| AF647 Anti-Human VISTA Antibody | BD Biosciences | Cat# 566670 | FC |
| APC-Cy7 Anti-Human CD45 Antibody | BD Biosciences | Cat# 557833 | FC |
| FITC Anti-Human CD68 Antibody | BioLegend | Cat# 333806 | ICFC |
| APC Anti-Human VISTA Antibody [B7H5DS8] | Thermo Fisher Scientific | Cat# 17-1088-42 | FC |
| MojoSort™ Human CD8 Nanobeads | BioLegend | Cat# 480108 | MACS^5^ |
| MojoSort™ Human CD14 Selection Kit | BioLegend | Cat# 480026 | MACS |
| Recombinant Human IL-2 (carrier-free) | BioLegend | Cat# 589102 | Stimulation |
| Ultra-LEAF Purified Anti-Human CD3 Antibody [OKT3] | BioLegend | Cat# 317326 | Stimulation |
| Ultra-LEAF Purified Anti-Human CD28 Antibody [CD28.2] | BioLegend | Cat# 302934 | Stimulation |
| AF647 Anti-Human CD68 Antibody | BD Biosciences | Cat# 562111 | ICFC |
| BV785 Anti-Human CD274 (PD-L1) Antibody | BioLegend | Cat# 329736 | FC |
| BV421 Anti-Human TNF-α Antibody | BioLegend | Cat# 502932 | ICFC |
| AF488 Anti-Human IL-12/IL-23 p40 Antibody | BioLegend | Cat# 501816 | ICFC |
| PE Anti-Human IL-10 Antibody | BD Biosciences | Cat# 562035 | ICFC |
| PE/Cyanine7 Anti-Human LAP (TGF-β1) Antibody | BioLegend | Cat# 349610 | FC |
| PE Anti-Human Arginase I Antibody | BioLegend | Cat# 369704 | ICFC |
| PerCP-Cy5.5 Anti-Human CD3 Antibody | BD Biosciences | Cat# 552852 | FC |
| FITC Anti-Human CD8 Antibody | BD Biosciences | Cat# 555366 | FC |
| APC Anti-Human IFN-γ Antibody | BD Biosciences | Cat# 551385 | ICFC |
| Horizon V450 Anti-Human Granzyme B Antibody | BD Biosciences | Cat# 561151 | ICFC |
| PE-Cyanine7 Anti-Human Perforin Antibody | Thermo Fisher Scientific | Cat# 25-9994-42 | ICFC |
| PerCP-Cy5.5 Mouse Anti-Human CD14 | BD Biosciences | 562692 | FC |
| PerCP-Cy5.5 Mouse Anti-Human CD19 | BD Biosciences | 561295 | FC |
| PerCP-Cy5.5 Mouse Anti-Human CD56 | BD Biosciences | 560842 | FC |
| BV421 Mouse Anti-Human CD123 | BD Biosciences | 563362 | FC |
| PE Mouse Anti-Human CD66b | BD Biosciences | 561650 | FC |
| PE-CF594 Rat Anti-CD11b | BD Biosciences | 562287 | FC |
| FITC Mouse Anti-Human HLA-DR | BD Biosciences | 555811 | FC |
| BV421 Mouse Anti-Human CD33 | BD Biosciences | 562854 | FC |
| PE-Cy7 Mouse Anti-Human CD16 | BD Biosciences | 560716 | FC |
| BV421 Mouse Anti-Human CD4 | BD Biosciences | 562424 | FC |
| BV421 Mouse Anti-Human CD3 | BD Biosciences | 563798 | FC |
| PE Mouse Anti-Human IL-12(p40/p70) | BD Biosciences | 559329 | FC |
| Arginase 1 Monoclonal Antibody | eBioscience | 12-3697-82 | FC |
| VISTA (D1L2G ™) XP ® Rabbit mAb | Cell Signaling Technology | 64953S | WB^6^ |
| GAPDH (14C10) Rabbit mAb | Cell Signaling Technology | 2118S | WB |
| Human VISTA/B7-H5/PD-1H Antibody [1011451] | R&D Systems | Cat# MAB71265-100 | Blockade |
| Mouse IgG_2B_ Isotype Control | R&D Systems | Cat# MAB004 | Isotype control |
| Camrelizumab (AiRuiKa^®^) | Suncadia Biopharmaceuticals | 201904001F | Blockade |
| Human IgG_4_ Isotype Control Recombinant Antibody | BioLegend | Cat# 403702 | Isotype control |
| AF700 Anti-Human CD8 Antibody | BioLegend | Cat# 344724 | FC |
| PerCP/Cyanine5.5 Anti-Human IFN-γ Antibody | BioLegend | Cat# 502525 | ICFC |
| AF647 Anti-Human/Mouse Granzyme B Antibody | BioLegend | Cat# 515406 | ICFC |
| PE Perforin Reagent Set | BD Biosciences | Cat# 556437 | ICFC |
| APC Anti-Human CD326 (EpCAM) Antibody | BioLegend | Cat# 324208 | FC |
| FITC Annexin V Apoptosis Detection Kit I | BD Biosciences | Cat# 556547 | FC |
| APC/Cyanine7 Anti-Human CD45 Antibody | BioLegend | Cat# 368516 | FACS^7^ |
| APC/Cyanine7 Mouse IgG_1_, κ Isotype Control | BioLegend | Cat# 400128 | Isotype control |
| FITC Anti-Human CD14 Antibody | BioLegend | Cat# 301804 | FACS |
| FITC Mouse IgG_2a_, κ Isotype Control | BioLegend | Cat# 400208 | Isotype control |
| AF647 Anti-Human VISTA Antibody | BD Biosciences | Cat# 566670 | FACS |
| AF647 Mouse IgG_1_, κ Isotype Control | BD Biosciences | Cat# 565571 | Isotype control |
| ^1^Immunohistochemistry;  ^2^Immunofluorescence;  ^3^Flow cytometry;  ^4^Intracellular staining for flow cytometry;  ^5^Magnetic activated cell sorting;  ^6^Western blot;  ^7^Fluorescence activated cell sorting. | | | |

| **Supplementary Table S3. List of software and algorithms used in the study.** | | |
| --- | --- | --- |
| **Software and algorithms** | **Source** | **Identifier** |
| IBM SPSS Statistics (version 21) | International Business Machines Corp | https://www.ibm.com/analytics/spss-statistics-software/ |
| GraphPad Prism (version 9.0.0) | GraphPad Software, LLC | https://www.graphpad.com/ |
| Stata (version 14.2) | StataCorp, LLC | https://www.stata.com/ |
| MedCalc Statistical Software (version 15.6.1) | MedCalc Software, BVBA | https://www.medcalc.org/ |
| FlowJo (version 10.0.7 R2) | Tree Star, Inc | https://www.flowjo.com/ |
| Image J (version 1.52a) | National Institutes of Health | https://imagej.nih.gov/ij/ |
| DMetrix ImageManager (version 1.0.0) | DMetrix, Inc | https://www.dmks.cn/ |
| CaseViewer (version 2.3) | 3DHISTEC, Ltd | https://www.3dhistech.com/ |
| Qlucore Omics Explorer (version 3.8) | Qlucore AB | https://www.qlucore.com/omics-explorer/ |
| Hiplot | Openbiox Community & Hiplot Team | https://hiplot.com.cn/ |
| scTIME Portal | Hong F, et al (2021). Single-Cell Analysis of the Pan-Cancer Immune Microenvironment and scTIME Portal. Cancer Immunol Res, 9(8):939-951. | http://sctime.sklehabc.com/#/home/ |
| Cutoff Finder | Budczies J, et a (2012). Cutoff Finder: a comprehensive and straightforward Web application enabling rapid biomarker cutoff optimization. PLoS One, 7(12):e51862. | https://molpathoheidelberg.shinyapps.io/CutoffFinder_v1/ |
| R (version 4.1.2) | The R Foundation for Statistical Computing | https://www.r-project.org/ |
| RStudio (version 2021.09.1+372) | RStudio, Inc | https://www.rstudio.com/ |
| timeROC R package (version 0.4) | Blanche P, Dartigues J, Jacqmin-Gadda H (2013). Estimating and Comparing time-dependent areas under receiver operating characteristic curves for censored event times with competing risks. Statistics in Medicine, 32(30):5381–5397 | https://CRAN.R-project.org/package=timeROC/ |
| survival R package (version 3.2-13) | Terry M. Therneau, Patricia M. Grambsch (2000). Modeling Survival Data: Extending the Cox Model. Springer, New York. ISBN 0-387-98784-3 | https://CRAN.R-project.org/package=survival/ |
| ggplot2 R package (version 3.3.5) | Wickham H (2016). ggplot2: Elegant Graphics for Data Analysis. Springer-Verlag New York. ISBN 978-3-319-24277-4 | https://ggplot2.tidyverse.org/ |
| DESeq2 (version 1.34.0) | Love, M.I., Huber, W., Anders, S. (2014) Moderated estimation of fold change and dispersion for RNA-seq data with DESeq2. Genome Biology, 15:550. | https://bioconductor.org/packages/3.14/bioc/bin/windows/contrib/4.1/DESeq2_1.34.0.zip |
| GSVA (version 1.42.0) | Hänzelmann S, Castelo R, Guinney J (2013). GSVA: gene set variation analysis for microarray and RNA-seq data. BMC Bioinformatics, 14:7. | http://127.0.0.1:25725/library/GSVA/doc/GSVA.html/ |

| **Supplementary Table S4. Real-time quantitative PCR primers.** | | |
| --- | --- | --- |
| **ID** | **Primer** | **Sequences (5' to 3')** |
| *VSIR* | Forward | ATTCCCTGTATGTCTGTCCCG |
| *VSIR* | Reverse | CTGCGGTACCACGTCTTGTAG |
| *GAPDH* | Forward | TGCACCACCAACTGCTTAGC |
| *GAPDH* | Reverse | GGCATGGACTGTGGTCATGAG |

| **Supplementary Table S5. List of genes selected for VISTA^+^ TAM signature.** |
| --- |
| *AC007848.2, AC009563.1, AC009041.3, AL118511.4, TUSC7, AC087289.2, MIR1-1HG-AS1, AC105445.1, AC068205.1, AP001628.2, PPDPFL, NCKAP5-AS2, AC136285.1, IGKV2D-29, Z97200.1, GFAP, MRGPRX3, AC015819.5, AL020994.1, LEUTX, AC092301.1, FAM242A, CYP4Z1, AC005908.3, LBX1, LINC02364, AP001020.3, CCDC183-AS1, AC095030.1, LCMT1-AS2, AL050404.1, LINC00668, AC092078.3, Z95114.2, LINC02167, AP002884.1, LINC02249, NTRK3-AS1, AC107373.1, AC093730.1, NUDT11, AP000755.2, AL033379.1, LINC01193, AF235103.1, AC092040.1, AC011322.1, IGKV1D-8, AL353611.1, AL589645.1, Z84486.1, BX088723.1, HTR3E-AS1, AC093503.2, AC130415.1, CCR8, AC080089.2, AL391427.1, AL137785.1, AC091868.2, S100G, AL513523.4, DNAJB8, AC024132.2, OPTC, MT4, GPRC6A, RESP18, AC073218.1, AC017099.2, OC90, AP000487.2, AC007491.1, IRGC, AL157938.4, GPIHBP1, PRAMEF33, LINC00354, OR8B3, AP003548.1, AC107398.3, CIB4, AC026320.3, TMEM262, AC114550.2, Z97353.2, AL442224.1, AL590560.5, CYP2A6, PGLYRP4, AC069133.1, TTTY4B, DLX1, LINC01570, AC022535.1, IGFN1, AL353704.2, AC115220.3, GPR37, AC005180.1, ZIC3, GOLGA6L22, LINC02111, AC107027.3, AC009623.3, AC084167.1, LINC02692, AC092813.2, ADGRA1, AC011466.2, PRICKLE2-AS3, DCD, AC002076.1, AC027688.1, LINC02214, AL358334.2, AC099552.2, AL358876.2, AL359258.2, AC009965.1, AL590705.2, AL161912.1, AL356056.1, LY6H, AC027779.1, AC100832.2, EPO, AC108752.1, AC007391.2, GDF6, SP5, AL365318.1, HOXD1, AL078587.1, LYPD8, AC092924.2, AL158835.1, AC093277.1, AL136307.1, AC004870.3, AC009407.1, CYP2W1, AC021220.2, LINC01858, AC004540.1, LINC00689, AL355053.1, AL513487.1, MPPED2-AS1, LINC01927, AC021355.1, LINC01687, AL133372.3, Z98949.3, LINC01228, AC092625.1, TAT, AC123595.1, AC026736.1, CCDC8, CCL19, AL355075.3, LINC00377, AC100839.2, AC114311.1, PZP, AC008033.3, AC009166.3, C1orf94, LINC01922, SLC25A47, AL137786.3, AC121161.2, AL356481.3, AC092994.1, AC092171.2, LINC00317, AP000265.1, SLC6A2, AC116345.1, Z97056.3, AP003170.4, AC018761.1, AC020558.6, AC145350.2, AL445213.2, NXF2B, AC068408.2, AC125603.1, ADAMTS17, ABCG4, GJD3, LINC00701, LINC02444, AL133244.2, AL049536.1, AC005909.1, AC079612.1, AC103719.1, AC008609.1, AL355312.2, AC105206.3, RGS13, AC067960.1, AL034550.2, AC140847.2, NCKAP5-AS1, AL121829.1, AC108935.1, AC020907.1, EMILIN3, CFAP58, AC099811.4, LINC01723, LINC00383, AP005436.2, AC018413.1, CDH6, LINC02003, AL157896.1, AL445668.1, LINC02835, AL121990.1, LINC00303, AC022405.1, LPP-AS1, AC104561.4, FAM240B, C20orf203, EIF2S3B, VSTM2A, ERFE, HS3ST6, AC096639.1, WFDC9, PSG4, FP671120.4, CTF1, FAM242D, AC023794.2, ADAD1, AL121894.1, CHODL-AS1, AC011524.2, AC022601.1, AC103691.1, PSG6, AC073052.2, CHST13, SMIM40, AC000099.1, AL606516.1, AC008060.2, AC242022.2, AC002074.2, CR392039.4, AC007795.1, STRA8, LINC02790, AC090018.2, LINC00604, AC011451.1, AC123786.3, AC087721.1, LINC01065, EDRF1-AS1, LILRA6, AL162394.1, AL162595.2, IGLL1, LINC01813, AC006062.1, AC005224.2, AC018541.1, CLEC6A, APOBEC3B-AS1, AL135908.1, AC092078.2, AC011389.1, AL096869.2, AL512634.1, AC099654.15, RDM1, AC124312.5, AL161753.1, SHISA4, AL109838.1, AC010991.1, LINC02594, ACCSL, AC003958.2, LINC01360, AL162171.2, AC018521.4, AL157413.1, AC105999.1, AC112236.3, C10orf90, AC007218.3, AC095050.1, AC090159.1, LINC01491, LINC02662, AC099499.1, AC024610.2, CYP3A7, TENT5D, AC026888.1, AC008572.1, AC021231.1, AC023644.1, AC110741.1, LINC00331, AC092723.4, RDH8, SGIP1, AL672277.1, AC103874.1, FCN1, AL137789.1, PDZRN3, AARD, FGF23, AL133216.1, WT1-AS, RIMS4, HTR2A, AC131254.1, AC013762.1, NAA11, AL445465.1, LEF1-AS1, AC134508.2, LINC01661, AC026470.2, FAM124B, UNC45B, AL049820.1, AC093911.1, AC093305.1, AL512356.5, LINC02805, AC009518.1, AC062039.1, DLX4, KCND1, EPDR1, PTHLH, AC079760.2, AC020704.1, AC016877.1, KLRB1, AL161646.1, AC010551.3, PTENP1-AS, AL357093.2, IZUMO1, AC013265.1, AL355592.1, AC010528.1, DIPK2B, AC027013.1, AC114498.1, CNTN4-AS2, CYP4F8, NMU, CCR4, OR10G3, AC133565.1, AC026991.2, MAMLD1, GNAT3, NTSR2, CRTAM, ZNF114, AC005154.4, NRK, AC007431.2, AC096570.1, AP004606.1, FOXN1, AP000820.2, AC068308.1, AC084346.1, AC062028.1, LINC01138, TBX18, EWSAT1, FAM27C, AC140481.2, GDF7, ANO3-AS1, TMEM233, AC011383.1, AC011586.1, SCARA5, AC026495.2, AL357833.1, AC007495.1, AC134043.2, LAMB1, AC009158.1, LHX2, AC068658.1, OPN1MW, AC090197.1, AL138999.2, LINC02309, TEX48, THBS2, AC008780.1, ADGRL3-AS1, LINC01280, CALB2, AL033530.1, TMEM212, TMPRSS6, AC010761.3, BCHE, MRGPRX2, LINC02016, KCNG1, MIR3976HG, LINC01020, AP000919.2, AC026992.2, AC069503.1, AC100768.1, RAPGEF4, CRHR1, AC005291.1, AL034417.4, ANO5, SERPINB9P1, AC008453.2, AL589642.1, GHRHR, LINC01933, AL356737.2, AL139231.1, ZNF679, LINC01229, LINC01243, AC114550.3, A1BG-AS1, OMD, LINC00885, MOG, AC099753.1, AC108721.2, AC105230.1, AC093425.1, NXF5, AC055733.2, LAMB4, LINC02098, FO393415.1, MPPED2, AC034213.1, XPNPEP2, AL512308.1, FGF9, DIAPH3-AS1, LCP2, ALOX15B, AP003100.2, PTMS, LAMA4, AC091180.2, AC009107.2, AL138686.1, LPO, AC244035.4, SPATC1, GPRC5D, AL161804.1, RAB39A, FAM53B-AS1, ZNF582, AC103855.3, AL133259.1, AC093843.1, FMO3, LINC02273, HLX-AS1, AL583804.1, ABCG1, AC007001.1, AC097459.1, KCNK10, AC091885.2, COLEC10, TMEM143, TM6SF2, AC093535.1, NHS, NPC1L1, AR, ISLR2, AC006013.1, AC007193.2, PAM, ZNF358, KNDC1, AL139381.1, LINC01844, SRD5A2, LINC01447, AL023882.1, SNHG5, RUNX3, CCDC175, AC087857.1, ZNF726, LRRN2, MEF2C-AS2, AL355312.3, PHKA1-AS1, AL109984.1, AC007533.1, KCNAB1, ATP8B1, NTM-AS1, LINC01756, LINC02234, MAP3K8, ATP1A2, AC138781.1, AL354794.1, HECW1, MPL, LMF1-AS1, AC068489.1, GOLGA8M, AL023755.1, LINC02067, LINC01695, ZC3H12B, AL451070.1, GSTM5, DPH6, AC005291.2, AC073288.1, PTCRA, FLNC-AS1, TMPRSS4, TP53TG5, CREB3L3, PODNL1, CHST8, LINC01121, TRIM67, DENND6A-DT, TBX15, PART1, MPDZ, LINC01389, AC087521.2, LOXL3, BLK, LMX1B, AL022238.2, DNAI2, ZNF845, KLHL42, FDXR, LAPTM4B, AL591895.1, ZNF614, TAP2, TUBE1, CIPC, DHODH, ZBED1, PAQR3, U73166.1, AC092139.1, LINC01176, AC130650.1, LRIG3, INKA2-AS1, LINC00310, AC012485.3, AL645728.1, MZF1-AS1, CD244, CXCL12, AC027279.1, AC092428.1, DNAJB5, AC008764.8, THAP9, AC117382.2, ZKSCAN2-DT, AC011445.2, TRAPPC3L, ZNF346-IT1, RSF1-IT1, PLPP6, KCNAB3, FIGNL1, VMO1, PRKAG2-AS1, AC017083.1, PCDHGA8, AC073476.3, HIST1H4B, TMCO6, AC099332.2, ST7-OT4, ALKBH6, NRROS, AC073389.1, LTC4S, GOLGA6L4, AC093297.2, GNGT2, AC005674.2, AC232271.1, AC023593.1, CEP83-DT, RAB42, AL121772.1, ZNF816, AL355353.1, AL356421.2, AL031728.1, TOLLIP-AS1, AC116158.2, TRAV6, AL031705.1, AC093462.1, FUT7, HCG9, AL137002.1, AC037459.2, SOWAHA, AC010478.1, AL049840.4, IQCF1, LINC02513, AC092332.1, AC007336.1, NHLRC1, AC016866.1, AC005256.1, AC036176.3, RAC3, TRIM74, IL36G, SRPK3* |
| Note: Six hundred and fifty-one differentially expressed genes (log_2_\|FC\| > 1, *P* < 0.05) were selected to construct VISTA^+^ TAM signature. |

**References**

1. Cao Y, Liu H, Li H, et al. Association of O6-Methylguanine-DNA Methyltransferase Protein Expression With Postoperative Prognosis and Adjuvant Chemotherapeutic Benefits Among Patients With Stage II or III Gastric Cancer. *JAMA Surgery* 2017; 152(11):e173120-e173120.

2. Cao Y, He H, Li R, et al. Latency-associated Peptide Identifies Immunoevasive Subtype Gastric Cancer With Poor Prognosis and Inferior Chemotherapeutic Responsiveness. *Annals of Surgery* 2022; 275(1):e163-e173.

3. Bass AJ, Thorsson V, Shmulevich I, et al. Comprehensive molecular characterization of gastric adenocarcinoma. *Nature* 2014; 513(7517):202-209.

4. Cristescu R, Lee J, Nebozhyn M, et al. Molecular analysis of gastric cancer identifies subtypes associated with distinct clinical outcomes. *Nature Medicine* 2015; 21(5):449-456.

5. Kim ST, Cristescu R, Bass AJ, et al. Comprehensive molecular characterization of clinical responses to PD-1 inhibition in metastatic gastric cancer. *Nature Medicine* 2018; 24(9):1449-1458.

6. Zhang P, Yang M, Zhang Y, et al. Dissecting the Single-Cell Transcriptome Network Underlying Gastric Premalignant Lesions and Early Gastric Cancer. *Cell Reports* 2019; 27(6):1934-1947.e5.

7. Cheng S, Li Z, Gao R, et al. A pan-cancer single-cell transcriptional atlas of tumor infiltrating myeloid cells. *Cell* 2021; 184(3):792-809.e23.

8. Budczies J, Klauschen F, Sinn BV, et al. Cutoff Finder: a comprehensive and straightforward Web application enabling rapid biomarker cutoff optimization. *PLoS One* 2012; 7(12):e51862.
